# *Vibrio splendidus* infection promotes circRNA-FGL1-regulated coelomocyte apoptosis via competitive binding to Myc with the deubiquitinase OTUB1 in *Apostichopus japonicus*

**DOI:** 10.1371/journal.ppat.1012463

**Published:** 2024-08-15

**Authors:** Ming Guo, Xin Li, Wenjun Tao, Fei Teng, Chenghua Li

**Affiliations:** 1 State Key Laboratory for Managing Biotic and Chemical Threats to the Quality and Safety of Agro-products, School of Marine Sciences, Ningbo University, Ningbo, China; 2 College of Mathematics and Computer, Jilin Normal University, Siping, Jilin, China; Uppsala University, SWEDEN

## Abstract

Circular RNAs (circRNAs) are involved in various physiological and pathological processes in both vertebrates and invertebrates. However, most studies on circRNAs have focused on their roles as endogenous competitive RNAs. Here, we report a novel function of circRNA derived from the Fibrinogen-like protein 1 gene (circ-FGL1) that inhibits coelomocyte apoptosis via competing with the deubiquitinase AjOTUB1 to bind AjMyc in *Apostichopus japonicus* during *Vibrio splendidus* infection. The results showed that circ-FGL1 is significantly downregulated in coelomocytes of *V*. *splendidus*-induced *A*. *japonicus* and negatively regulates coelomocyte apoptosis through the AjBax-AjCyt c pathway. Mechanistically, the deubiquitinase AjOTUB1 and circ-FGL1 could interact with the transcription factor protein AjMyc in the same region with circ-FGL1/AjMyc having greater affinity. Under normal conditions, high levels of circ-FGL1 bind directly to AjMyc, inhibiting the deubiquitylation of AjMyc by AjOTUB1 and leading to the degradation of AjMyc. After *V*. *splendidus* infection, AjMyc disassociates from the depressed expression of circ-FGL1, promoting its deubiquitylation by binding to the induced deubiquitinase AjOTUB1 to inhibit its degradation. AjMyc is then transferred to the nucleus and promotes the transcription of AjCyt c and AjBax to induce coelomocyte apoptosis. The new finding will expand our present outstanding on the functional role of circRNAs and suggest new therapeutic targets for the treatment of echinoderms during bacterial invasion.

## Author summary

*Apostichopus japonicus*, a sea cucumber belonging to the phylum Echinodermata, represents an exceptional model for scientific research due to its unique evolutionary position between invertebrates and vertebrates. As a class rich in active substances with significant nutritional and medicinal value, sea cucumbers face threats from various pathogens, especially skin ulcer syndrome infected by *Vibrio splendidus*, which has caused substantial economic losses in *A*. *japonicus* aquaculture. *A*. *japonicus* counteracts pathogen invasion through innate immunity and programmed cell death. Apoptosis, in particular, is the predominant mechanism for regulating cell death in echinoderms and has important implications for pathogen-induced immune homeostasis. Here, we identified circ-FGL1 as an antiapoptotic circRNA and found that circ-FGL1 interacts preferentially with the AjMyc protein, leading to the promotion of AjMyc degradation via ubiquitination under normal conditions. After *V*. *splendidus* infection, induced AjOTUB1, a deubiquitinase, interacts with AjMyc at the same region as circ-FGL1 and facilitates the deubiquitination of AjMyc. AjMyc is then transferred to the nucleus, where it promotes the transcription of AjCyt c/AjBax, ultimately inducing coelomocyte apoptosis. Our study offers potential new therapeutic targets for treating echinoderms during the bacterial invasion.

## Introduction

Echinoderms, lack adaptive immunity and rely solely on innate immunity to resist pathogen infection. Despite this limitation, they possess a repertoire of conserved immune regulatory genes and transduction pathways that enable them to cope with pathogen infection and maintain immune homeostasis [1–3]. The coelomocytes of echinoderms exhibit functions similar to those of vertebrate hemocytes and play crucial roles in immune defense, including apoptosis, autophagy, pyrosis, necrosis, *etc*. [4]. Apoptosis, in particular, is the predominant mechanism for regulating cell death in echinoderms and has important implications for pathogen-induced immune homeostasis [5,6]. In humans, resistance to apoptosis is a hallmark of cancer and can lead to treatment failure [7]. Additionally, inhibitors of apoptosis molecules, such as MCL1 inhibitors and Bcl-2 inhibitors, have shown applications in clinical settings [8,9]. Although many apoptosis-related molecules have been identified and characterized in echinoderms [10–12], the underlying mechanisms and potential applications of these molecules remain largely unexplored. Therefore, investigating the functional mechanisms of proapoptotic and antiapoptotic molecules is crucial for developing strategies to combat pathogen invasion in echinoderms.

Over the past few decades, noncoding RNAs (ncRNAs) have emerged as key players in a wide range of biological processes in both vertebrates and invertebrates due to advancements in high-throughput sequencing technology [13,14]. Specifically, circular RNAs (circRNAs), a unique subclass of endogenous ncRNAs generated by pre-mRNAs via back-splicing of covalently linked 5’- and 3’-ends without the poly(A) tail, have attracted significant attention. Unlike linear RNAs, circRNAs possess closed-loop structures that render them resistant to degradation by exonucleases. Recent research has revealed that circRNAs exhibit multiple functions, including serving as miRNA sponges, regulating gene transcription, interacting with RNA-binding proteins, and modulating protein translation [15,16]. Moreover, circRNAs were also reported to they can influence the protein stability of transcriptional regulators through the ubiquitin-proteasome system [17,18]. Our previous studies in *Apostichopus japonicus* demonstrated the involvement of circRNAs in the progression of skin ulcer syndrome (SUS) caused by *Vibrio splendidus* infection [3,6,19]. However, most existing research on circRNAs has focused primarily on their function as endogenous competitive RNAs. Therefore, additional investigations are needed to elucidate the underlying mechanisms by which circRNAs modulate gene transcriptional regulation in pathogen-induced echinoderms.

The myelocytomatosis viral oncogene (Myc) belongs to the basic helix-loop-helix-leucine (bHLH) zipper family and serves as a transcription factor that plays pivotal roles in cell growth, death, differentiation, metabolism, and apoptosis [20]. Previous research has demonstrated a strong correlation between the occurrence and progression of various diseases and apoptosis, with Myc being frequently overexpressed and mutated [21,22]. Studies have revealed that the expression of Myc initiates a series of apoptotic processes by stimulating caspase activity [23]. Caspase 3, an apoptotic effector, has been found to undergo cleavage into active subunits during the early stages of Myc-induced apoptosis [24]. The bHLH domain and functional transactivation domain of Myc provide a solid foundation for the involvement of Myc target genes in Myc-mediated apoptosis [25,26]. As a transcription factor, Myc typically binds to a common DNA sequence known as the E-box (CANNTG) within the promoter and/or enhancer region to regulate cell apoptosis [27]. Research has indicated that Bax, one of the essential proteins for initiating the apoptosis cascade, is also a downstream effector required for Myc-mediated apoptosis, including in *A*. *japonicus* [28–30]. In the presence of Myc, elevated expression of Bax is observed in the mitochondria of apoptotic cells [31]. Furthermore, previous studies have confirmed the involvement of posttranslational modification of the Myc protein in apoptosis [17]. It has also been reported that the stability and transcriptional activity of the Myc protein can be regulated by noncoding RNAs, which in turn mediate cell apoptosis [17,32].

*Apostichopus japonicus*, a sea cucumber belonging to the phylum Echinodermata, represents an exceptional model for scientific research due to its unique evolutionary position between invertebrates and vertebrates. In addition, *V*. *splendidus*-induced SUS is a prevalent disease in the breeding of *A*. *japonicus*, resulting in over 90% mortality and significant economic losses [33]. In this study, we identified circ-FGL1 as an antiapoptotic circRNA and found that this circRNA interacts preferentially with the AjMyc protein, leading to the promotion of AjMyc degradation via ubiquitination. Additionally, we discovered that AjOTUB1, a deubiquitinase, interacts with AjMyc at the same region as circ-FGL1 and facilitates the deubiquitination of AjMyc. Our study revealed a previously unknown mechanism through which *V*. *splendidus* infection promotes coelomocyte apoptosis regulated by circRNA-FGL1 via AjMyc deubiquitination by AjOTUB1 in *A*. *japonicus*. This novel finding expands our current understanding of the functional role of circRNAs and provides new therapeutic targets for mitigating bacterial invasion in echinoderms.

## Materials and methods

### Ethics statement

The sea cucumbers, *Apostichopus japonicus*, were commercially cultured, and all experiments were conducted in accordance with the recommendations in the Guide for the Care and Use of Laboratory Animals of the National Institutes of Health. The study protocol was approved by the Experimental Animal Ethics Committee of Ningbo University, China (No. NBU-ES-2021-11180).

### Animals, challenge experiments, and sampling

Healthy *A*. *japonicus* sea cucumbers (~ 110 ± 5 g) were procured from Xinyulong Aquaculture Company (Dalian City, China) and raised in natural seawater for five days before treatment. For an immune challenge, *A*. *japonicus* were bathed with a final concentration of 10^7^ CFU of *V*. *splendidus*. Tissue samples, including coelomocytes, muscle, intestine, tentacle, and respiratory tree, were collected at 0, 6, 12, 24, 48, and 72 h posttreatment.

### Cell culture and treatment

Sea cucumber coelomocytes were obtained from the coelomic fluids of healthy sea cucumbers using precooled anticoagulant (0.02 M EGTA, 0.48 M NaCl, 0.019 M KCl, 0.068 M Tris–HCl, pH 7.6). The cells were centrifuged at 800 × g at 4°C for 5 min and then incubated in Leibovitz’s L-15 medium supplemented with streptomycin sulfate (100 mg mL^−1^) and penicillin (100 U/mL^−1^) at 16°C. For lipopolysaccharide (LPS) stimulation, the incubated coelomocytes were exposed to LPS for 0, 3, 6, 12, or 24 h at a final concentration of 1 mg mL^−1^. The HEK 293T cell line was purchased from Biobw Company (Beijing, China) and cultured in DMEM supplemented with 10% FBS and 5% CO_2_ at 37°C.

### Construction of expression vectors or plasmids

Based on the potential binding regions and sites between circ-FGL1 and AjMyc and between AjMyc and the OTU domain-containing ubiquitin aldehyde-binding protein Otubain1 of *A*. *japonicus* (AjOTUB1), overexpression vectors for circ-FGL1 and AjMyc, including circ-FGL1-f1 (1100–1500 bp), circ-FGL1-f2 (3100–3400 bp), AjMyc-f1 (1–431 amino acid (aa)), AjMyc-f2 (201–260 aa), and AjMyc-f3 (300–407 aa), were constructed in the pcDNA3.1-CMV-circRNA-Zsgreen (Haijihaoge Biotechnology, Shanghai, China) or pcDNA3.1–3 × FLAG-C vector. Overexpression vectors for AjOTUB1, including AjOTUB1-f1 (1–266 aa) and AjOTUB1-f2 (110–210 aa), were also constructed in the pcDNA3.1-3-EGFP vector. Furthermore, overexpression vectors for circ-FGL1, AjMyc, and AjOTUB1 mutants were constructed by Hanheng Biotechnology (Shanghai, China). The recombinant plasmids were extracted, and the endotoxins were removed using a Plasmid DNA Maxi Purification Kit (Med Chem Express, USA). For subsequent functional analysis, endotoxin-free plasmids were transfected into coelomocytes or HEK 293T cells. The specific primers and restriction endonucleases used for expression vector construction in this study are listed in S1 Table.

### Cell transfection

Transient transfection of HEK 293T cells or coelomocytes was conducted using overexpression vectors for circ-FGL1, AjOTUB1, or AjMyc, along with their WT and MUT variants (2 μg). Small interfering RNAs (siRNAs) targeting circ-FGL1, AjMyc, AjOTUB1, Ajcaspase 3, AjBax, and AjCyt c (20 μM), as well as their respective controls, were also transfected into cells. The transfections were carried out in 24-well microplates using Lipofectamine 6000 transfection reagent (Beyotime, Shanghai, China) according to the manufacturer’s instructions. The specific siRNAs used were synthesized by GenePharma, Shanghai, China (S1 Table).

### Bacterial invasion assay

The number of *V*. *splendidus* invading coelomocytes was measured using the plate counting method [19]. Sea cucumber coelomocytes were treated with siNC or si-circ-1 for 24 h prior to infection. The siRNA-treated coelomocytes were then injected with *V*. *splendidus* (1 × 10^7^ cells/mL) for 12 h. The infected coelomocytes were collected, lysed, diluted 1000-fold, and inoculated onto 2216E agar plates in triplicate. The colonies were counted after overnight incubation at 28°C.

### Rapid application of full-length AjOTUB1 cDNA

Partial AjOTUB1 core sequences were obtained from the *A*. *japonicus* transcriptome database [34]. Total RNA was extracted using RNAiso plus, and reverse transcription was performed using the 1st cDNA Synthesis Kit. The full-length cDNA sequence of AjOTUB1 was obtained through rapid amplification of cDNA ends (RACE)-PCR using the RACE 5’/3’ Kit (Takara, Japan) following the manufacturer’s instructions. The specific primers used to amplify the AjOTUB1 products are listed in S1 Table.

### Identification of binding sites

The interactions between circ-FGL1 and AjMyc were predicted using the catRAPID database (http://service.tartaglialab.com/page/catrapid_group). Specifically, the catRAPID omics module simulated interactions between molecules (proteins/transcripts) and reference sets (transcripts/nucleotide-binding proteins) from model organisms [35]. After an initial screening to identify potentially interacting RNAs and proteins, we segmented these molecules into fragments using the catRAPID fragment module [36]. This segmentation allowed for the prediction of specific binding sites between the RNAs and proteins, providing detailed binding information. To gain insight into these interactions at the molecular level, we performed structural prediction and docking experiments on the macromolecules [37]. Next, we predicted the secondary structure and thermodynamic properties of circ-FGL1 using the RNAfold web server (http://rna.tbi.univie.ac.at/cgi-bin/RNAWebSuite/RNAfold.cgi) [38,39]. The tertiary structures of circ-FGL1 were then modeled using 3D-RNA, whereas the structures of AjMyc were predicted using AlphaFold2 [40]. To elucidate the atomic-level mechanisms of these interactions, we performed protein-RNA docking using the NPDock server (https://genesilico.pl/NPDock/submit). This process generated 20,000 models to identify optimal binding modes. NPDock combines GRAMM algorithms for docking, scoring, and clustering, with the refinement of the top-scoring complex [41]. Finally, we utilized PyMOL open-source software to visualize the atomic details of the predicted binding interfaces. Through this structural modeling, we obtained detailed insights into the interaction mechanisms at the molecular level. For protein-protein docking (between AjOTUB1 and AjMyc), we utilized the ZDOCK server (https://zdock.umassmed.edu/). The highest-scoring predicted complex was selected for further analysis. Residues participating in intermolecular interactions were identified by inspecting the model in PyMOL open-source software.

### RNA extraction and quantitative reverse transcription-polymerase reaction (qRT-PCR)

Total RNA extraction, cDNA reverse transcription, and PCR analysis were conducted as previously described [6]. In some cases, cytoplasmic and nuclear RNA was extracted from coelomocytes using a Cytoplasmic & Nuclear RNA Purification Kit (Norgen Biotek Corp, Canada). The Applied Biosystems Q3 PCR system was used for gene analysis, and the 2^-ΔΔCT^ method was employed to analyze the relative expression changes of genes in this study [42]. The primers used for qRT–PCR can be found in S1 Table.

### Flow cytometry

Apoptosis assays were conducted using propidium iodide (PI) and an Annexin V-FITC apoptosis detection kit (Beyotime, Beijing, China) following the manufacturer’s protocol as previously described [6]. Briefly, sea cucumber coelomocytes were treated with circ-FGL1, Ajcaspase 3, AjCyt c, siAjMyc, siAjBax (20 μM), *V*. *splendidus* (10^7^ CFU mL^−1^), LPS (0.1 μg/μL), or their respective controls. The cells were then incubated in a binding buffer and treated with Annexin V-FITC and PI at room temperature for 20–25 min in the dark. Flow cytometry (Agilent NovoCyte, Santa Clara, USA) was used to detect and analyze the levels of apoptosis. The coelomocytes in the Q2-2 and Q2-4 regions represented early and late apoptotic cells, respectively. The apoptosis rates were calculated by dividing the coelomocyte numbers in the regions, such that (Q2-2 + Q2-4)/(Q2-1 + Q2-2 + Q2-3 + Q2-4).

### Immunoprecipitation (IP), mass spectrometry (MS), and Western blotting

Following treatment or transfection, coelomocytes or HEK 293T cells were subjected to protein sampling using RIPA lysis buffer (Beyotime, Beijing, China) and protein quantification using a BCA protein assay kit (Beyotime, Beijing, China). The cytoplasmic and nuclear proteins from coelomocytes were extracted using the NE-PER Nuclear and Cytoplasmic Extraction Kit (Thermo Fisher, USA). In other cases, proteins for ubiquitination and co-IP detection were obtained using a Cell Lysis Buffer for Western blotting and Immunoprecipitation (IP) (Beyotime, Beijing, China). To detect proteins that interact with AjMyc, co-IP and MS were performed. The MS data of proteins immunoprecipitated by anti-AjMyc antibodies or pre-immune serum were deposited in the ProteomeXchange Consortium (https://proteomecentral.proteomexchange.org) via the iProX partner repository [43,44] with the dataset identifier PXD051548. For the western blotting assays, protein samples were separated on 12% or 15% SDS–PAGE gels and then subjected to nitrocellulose or polyvinylidene fluoride membrane electrophoresis. After incubation with primary antibodies, including anti-AjMyc mouse antibody (1:1000, GenScript Technology), anti-AjOTUB1 rabbit antibody (1:1000, GenScript Technology), anti-AjBax rabbit antibody (1:1000, GenScript Technology), anti-Ajcaspase 3 rabbit antibody (1:1000, A0214, ABclonal), anti-AjCyt C rabbit antibody (1:500, GenScript Technology), anti-Ajβ-actin mouse antibody (1:5000, M20027, Abmart), anti-Histone H3 rabbit antibody (1:2000, P30266, Abmart), anti-Hsβ-actin mouse antibody (1:5000, 81115-1-RR, Proteintech), anti-AjFGL1 rabbit antibody (1:500, GenScript Technology), HRP-labeled anti-FLAG mouse antibody (1:5000, HRP-66008, Proteintech), and HRP-labeled anti-GFP rabbit antibody (1:2000, AE031, ABclonal), the membranes were incubated with HRP-labeled goat anti-rabbit IgG (1:8000, D110058-0100, Sangon Technology) or mouse IgG (1:8000, D110087-0100, Sangon Technology). Finally, the signals were measured using a chemiluminescence system (Bio-Rad, USA) after washing with PBST. The bands were quantitatively analyzed using ImageJ software, and the gray values are shown as the statistical analysis of three independent experiments.

### RNase R treatment

Total RNA was extracted, quantified, and incubated with RNase R (3 U/μg, Epicenter, USA) at 37°C for 30 min. Subsequently, the RNA was reverse transcribed, reverse transcribed with specific divergent primers for circ-FGL1 (S1 Table), and subjected to RT-PCR, qRT-PCR, and nucleic acid electrophoresis analysis.

### RNA immunoprecipitation (RIP) assay

An RNA RIP assay was conducted using the Magna RIP RNA-Binding Protein Immunoprecipitation Kit (Merck Millipore, USA) to evaluate the expression of circRNAs. Initially, the unrelated proteins in coelomocytes or HEK 293T cells were treated with protein A + G agarose beads (Beyotime, Beijing, China). The protein extracts were then incubated with various antibodies, including anti-AjMyc mouse antibody (1:1000, GenScript Technology), anti-AjOTUB1 rabbit antibody (1:1000, GenScript Technology), Argonaute 2 (AGO2) rabbit antibody (1:500, Boster Biological Technology, China), HRP-labeled anti-FLAG mouse antibody (1:5000, HRP-66008, Proteintech), HRP-labeled anti-GFP rabbit antibody (1:2000, AE031, ABclonal), and pre-immune serum. This was followed by incubation with protein A + G beads for 18 h at 4°C. Subsequently, proteinase K was used to remove unrelated proteins in the RNA–protein complexes. Finally, the RNA was sampled for further RT-PCR or qRT-PCR detection.

### RNA fluorescence in situ hybridization (FISH) and immunofluorescence assay

A FISH assay was carried out using a FISH Tag RNA Kit (F32954, Thermo Fisher, USA) following the manufacturer’s protocol. Specifically, probes targeting circ-FGL1 were synthesized and labeled with Alexa Fluor 594 fluorescent dye, whereas DAPI was used to stain the nuclei. Immunofluorescence staining was performed as previously described [3]. Coelomocytes were fixed, permeabilized, blocked, and incubated with primary antibodies, including anti-AjMyc mouse antibody (1:1000, GenScript Technology) or anti-AjOTUB1 rabbit antibody (1:1000, GenScript Technology), overnight at 4°C. Afterward, the cells were incubated with Cy3-labeled goat anti-rabbit IgG (1:5000, A0516, Beyotime, Beijing, China) and FITC-labeled goat anti-mouse IgG (1:5000, A0568, Beyotime, Beijing, China). Finally, the cells were observed using a laser confocal microscope (Leica, Germany).

### RNA sequencing (RNA-seq)

Sea cucumbers were treated with circ-FGL1 siRNA and its control siNC (20 μM). RNA-seq and data analysis were performed by Novogene Company (Beijing, China). Paired-end libraries were constructed using the NEBNext Ultra RNA Library Prep Kit, and the sequencing data were analyzed using an Illumina NovaSeq 6000 platform. Genes with significantly different expressions were defined based on the following criteria: a fold change greater than 2 and a *P* value less than 0.05. The RNA-seq data can be found in the GEO database under BioProject number PRJNA1101092 (accession no. SRR28709429 and SRR28709430).

### Pull-down assay and MS analysis

For RNA pull-down assays, biotinylated circ-FGL1, circ-FGL1-f1, and circ-FGL1-f2 were synthesized by GenePharma Biotech (Shanghai, China). The sequences of these probes were specifically complementary to the head-to-tail splicing region, and pull-down assays were conducted as described previously [45]. Briefly, the probes (200 pmol) were incubated with streptavidin magnetic beads (Thermo Fisher Scientific, USA) at 4°C overnight to obtain probe-coated beads. A total of 1 × 10^7^ coelomocytes, intestines, or HEK 293T cells were harvested, lysed, and sonicated to generate cell lysates. The cell lysates were then incubated with the probe-coated beads at 4°C overnight with rotation. After washing, the RNA-binding proteins were eluted for subsequent analysis. For MS analysis, the RNA complexes were subjected to SDS–PAGE, and differentially expressed proteins were identified by BioMarker Biotech Co., Ltd. The MS data were deposited in the ProteomeXchange Consortium (https://proteomecentral.proteomexchange.org) via the iProX partner repository [43,44] with the dataset identifier PXD051546. For protein pull-down assays, FLAG-tagged AjMyc protein (rAjMyc-f1), EGFP-tagged AjOTUB1 protein (rAjOTUB1-f1), EGFP-tagged AjOTUB1 mutant protein (AjOTUB1-f1-mut), circ-FGL1-f1, and circ-FGL1-f1-mut were prepared and purified. rAjMyc-f1 (1 μg) was immobilized on FLAG resin, and then rAjOTUB1-f1 (3 μg), circ-FGL1-f1 (3 μg) (Group A), circ-FGL1-f1 (3 μg), rAjOTUB1-f1-mut (3 μg) (Group B), circ-FGL1-f1-mut (3 μg) and rAjOTUB1-f1 (3 μg) (Group C) were mixed and added to the FLAG-AjMyc-conjugated resin overnight at 4°C. The resin was washed and eluted with FLAG elution buffer to obtain protein-RNA and protein-protein complexes. The eluate was then collected for qRT–PCR and co-IP analysis.

### *In vivo* ubiquitination assay

A ubiquitination assay was conducted in si-circ-1-, siAjOTUB1-, or siNC-treated coelomocytes or HEK 293T cells for 24 h. The cells were then treated with MG132 (20 mM, Boston, MA, USA) or N-ethylmaleimide (NEM, 5 μM) for 6 h before collection. The ubiquitinated AjMyc in the cell lysate was isolated using an anti-AjMyc antibody (1:1000, GenScript Technology), and the level of endogenous AjMyc after treatment was measured using a chemiluminescence system (Bio-Rad, USA).

### Cycloheximide (CHX) chase assay

A CHX chase assay was conducted to investigate the half-life of AjMyc. Coelomocytes were treated with si-circ-1 or siAjOTUB1 for 24 h and then treated with CHX (100 μg/mL, MCE, Lexington, MA, USA) or NEM (5 μM) for 0, 3, 6, 9, or 12 h. Coelomocytes were sampled at the indicated time points, and total protein lysates were used to detect AjMyc expression by western blotting. Semiquantitative analysis of AjMyc protein expression was performed using ImageJ software.

### Statistical analysis

Student’s t-test and ANOVA were used for statistical analysis in this study. The results were obtained from at least three independent experiments, and the data are presented as the means ± SDs according to the 2^-ΔΔCT^ method [42]. For the statistical analysis, a significant difference was indicated as *p* < 0.05.

## Results

### circ-FGL1 is downregulated in *V*. *splendidus*-challenged immune tissues of sea cucumbers

**Fig 1:**
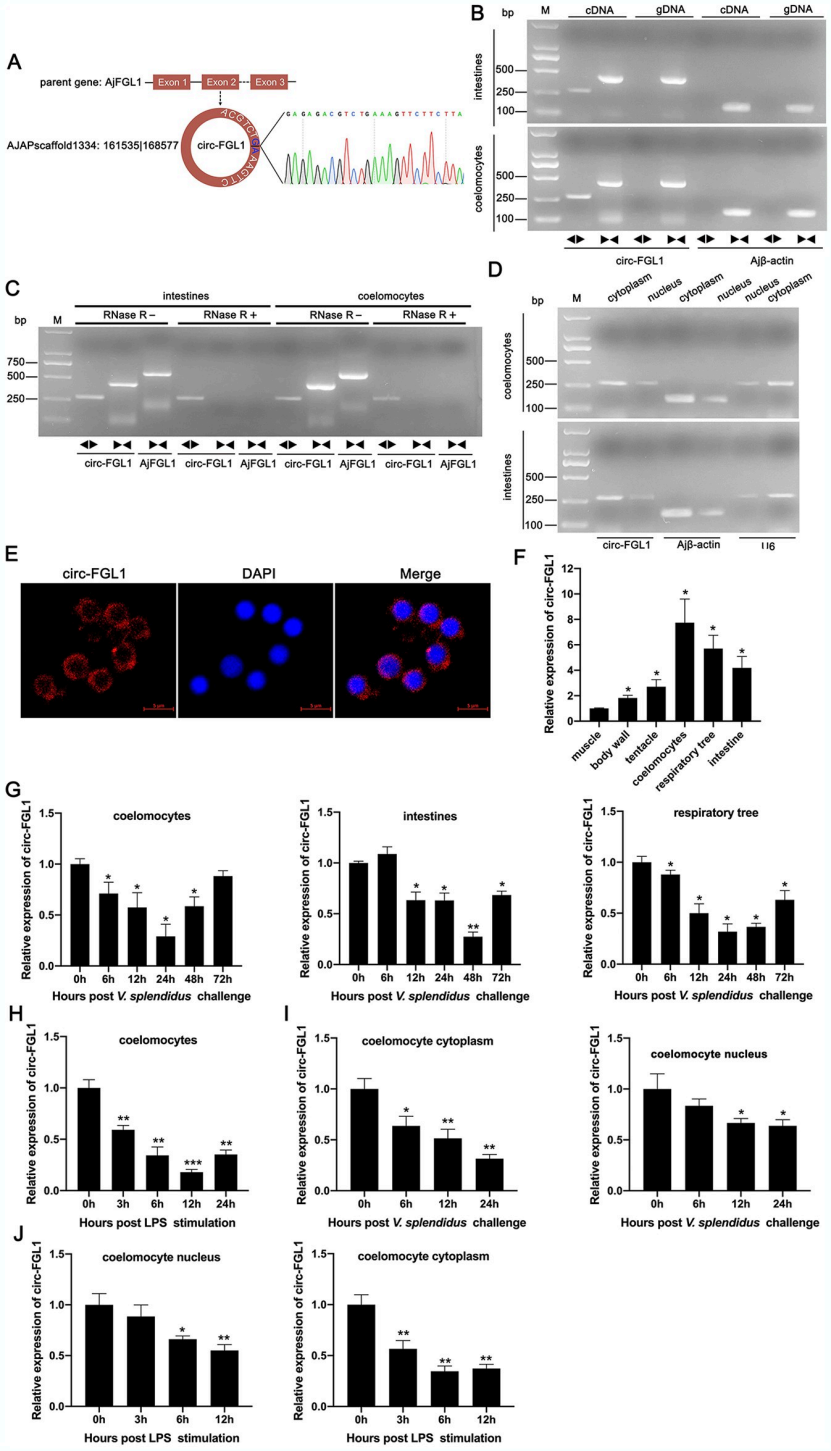
circ-FGL1 validation and expression in *A*. *japonicus* coelomocytes and tissues. (**A**) Schematic illustration showing the circularization form of circ-FGL1 derived from its host gene AjFGL1. The existence of circ-FGL1 was further verified by RT-PCR and Sanger sequencing. The blue words (G and A) indicate the head-to-tail splicing sites of cir-FGL1. (**B**) circ-FGL1 was detected in the intestine and coelomocytes by RT-PCR with divergent primers and convergent primers. circ-FGL1 was amplified from cDNA but not in gDNA with divergent primers. Ajβ-actin served as a negative control. (**C**) The expression levels of circ-FGL1 and AjFGL1 mRNA in the intestine and coelomocytes treated with or without RNase R were measured by RT-PCR. (**D**) The expression of circ-FGL1 in the cytoplasm and nucleus of the intestine and coelomocytes was detected by RT-PCR. Ajβ-actin and U6 served as the controls. (**E**) RNA FISH showed that Alexa Fluor 594-labeled circ-FGL1 was mainly localized in the cytoplasm. Nuclei were stained with DAPI. Scale bar: 5 μm. (**F**) circ-FGL1 expression in muscles, body walls, tentacles, coelomocytes, respiratory trees, and intestines was measured by qRT-PCR. (**G**) The expression of circ-FGL1 in coelomocytes, the intestine, and the respiratory tree of *V*. *splendidus*-challenged *A*. *japonicus* was detected by qRT-PCR. (**H**) circ-FGL1 expression in LPS-stimulated primary coelomocytes was measured by qRT-PCR. (**I**, **J**) The expression of circ-FGL1 in the cytoplasm and nucleus of coelomocytes post-*V*. *splendidus* challenge (I) or LPS stimulation (J) was detected by qRT-PCR. Ajβ-actin served as the negative control. The data are represented as the mean ± SD; n = 3. *P < 0.05, **P < 0.01, ***P < 0.001 indicate the significant differences.

To explore the roles of circRNAs in SUS-diseased sea cucumbers, we detected circRNA expression in coelomocytes of healthy or *V*. *splendidus*-challenged sea cucumbers, based on our previous RNA-seq sequencing information [46]. We identified several circRNAs, including a downregulated circRNA derived from the fibrinogen-like protein 1 gene (circ-FGL1, AJAPscaffold1334:161535|168577). circ-FGL1 is originates from the *A*. *japonicus* FGL1 (AjFGL1) gene and contains one exon and one intron, as annotated in GenBank accession no. PP690939) (Fig 1A). To confirm the existence of circ-FGL1 in *A*. *japonicus*, we employed RT-PCR with specific divergent and convergent primers to examine the head-to-tail splicing of circ-FGL1. Our results showed that the divergent primers for circ-FGL1, but not Ajβ-actin, amplified a specific PCR product in both coelomocytes and intestines (Fig 1B). The amplification products of circ-FGL1 with specific divergent primers were further confirmed by Sanger sequencing (Fig 1A). Considering the circular structure of circRNAs, they are more stable and resistant to degradation by RNase R than linear RNAs. qPCR analysis revealed that circ-FGL1 in total RNA from coelomocytes and intestines remained intact after RNase R treatment, whereas the mRNA levels of AjFGL1 decreased sharply under the same conditions (Fig 1C). Then, we conducted a cytoplasmic nuclear fractionation assay to detect the distribution of circ-FGL1 in untreated coelomocytes, and the results showed that circ-FGL1 was located in both the cytoplasm and nucleus (Fig 1D). This result was further confirmed by RNA FISH assay through a specific fluorescent probe targeting circ-FGL1 (Fig 1E). Subsequently, we investigated the expression of circ-FGL1 in various tissues of healthy and *V*. *splendidus*- or LPS-treated sea cucumbers. In healthy sea cucumbers, circ-FGL1 exhibited high expression levels in coelomocytes, the respiratory tree, and the intestines, followed by the tentacle and the body wall, with lower expression in muscles (Fig 1F). In contrast, we observed that the levels of circ-FGL1 were significantly downregulated in the coelomocytes, respiratory tree, and intestines of *V*. *splendius*-challenged sea cucumbers (Fig 1G). This downregulation was also confirmed in LPS-exposed cultured primary coelomocytes (Fig 1H). Considering the predominant cytoplasmic and nuclear distribution of circ-FGL1, we further investigated its expression in the cytoplasm and nucleus following *V*. *splendidus* challenge or LPS stimulation. The results revealed that significant decrease in circ-FGL1 expression in both the cytoplasm and nucleus, regardless of the conditions of *V*. *splendidus* challenge (Fig 1I) or LPS stimulation (Fig 1J). Taken together, these findings reveal that circ-FGL1 is downregulated in tissues challenged with pathogen, and that circ-FGL1 is localized to the cytoplasm and nucleus.

### Decreased circ-FGL1 promotes coelomocyte apoptosis in response to pathogen infection

**Fig 2:**
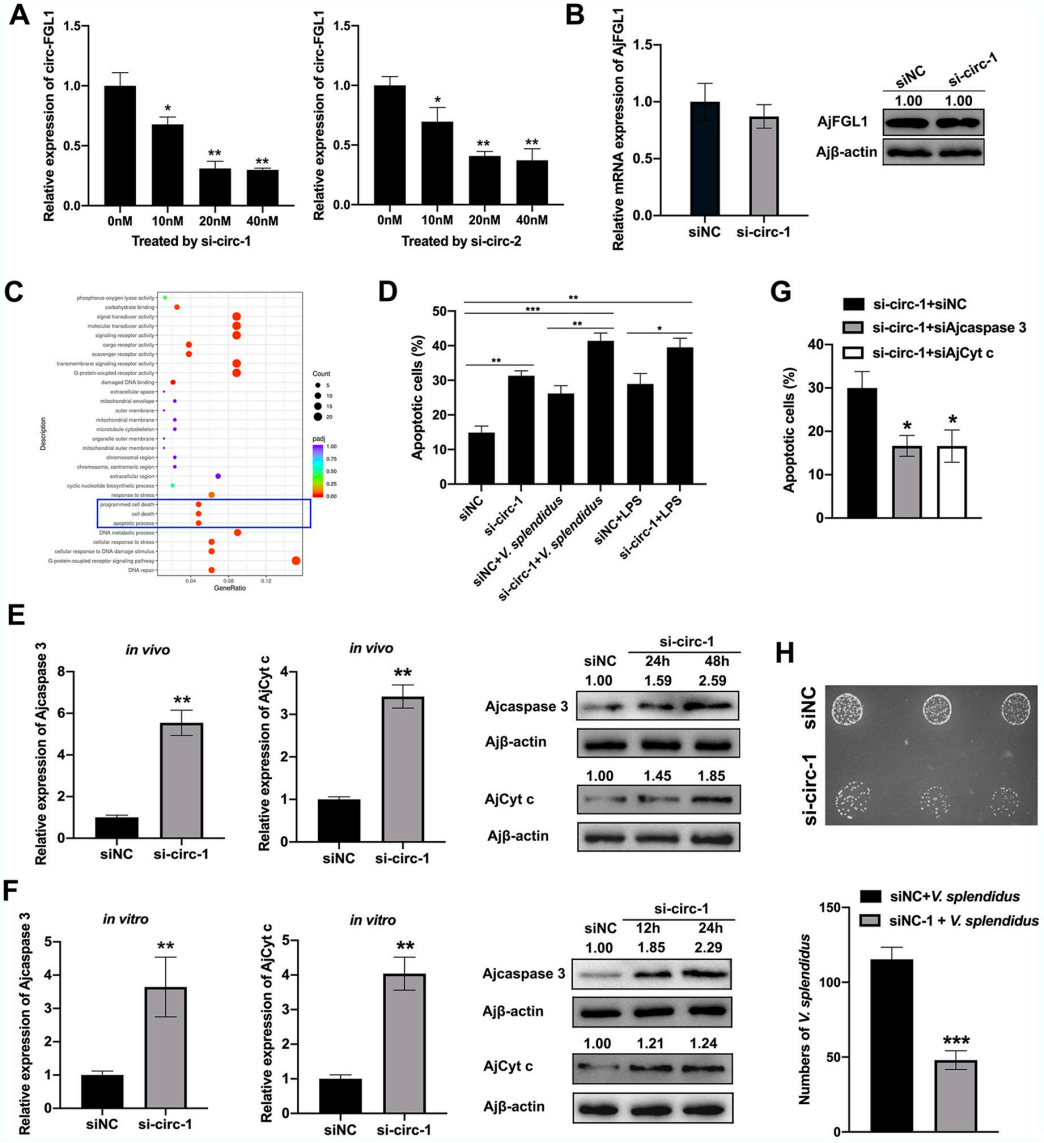
circ-FGL1 inhibits the apoptosis signaling pathway in *A*. *japonicus*. (**A**) circ-FGL1 expression in coelomocytes post treatment with different concentrations of circ-FGL1 (si-circ-1 or si-circ-2) or control siRNAs was measured by qRT-PCR. (**B**) AjFGL1 mRNA and protein expression levels in coelomocytes post siNC or si-circ-1 treatment were detected by qRT-PCR and western blotting, respectively. (**C**) Pathways enriched by GO on the basis of RNA-seq data of coelomocytes treated with circ-FGL1 siRNA and the siNC control. The enriched apoptotic pathways are marked in the blue box. (**D**) Coelomocyte apoptosis was detected after circ-FGL1 interference for 24 h, followed by *V*. *splendidus* challenge or LPS exposure for another 24 h by flow cytometry. (**E**, **F**) The mRNA and protein expression levels of Ajcaspase 3 and AjCyt c in si-circ-1- or siNC-treated *A*. *japonicus* coelomocytes (E), or primary cultured coelomocytes (F) were detected by qRT-PCR and western blotting, respectively. (**G**) Ajcaspase 3 and AjCyt c function in circ-FGL1-silenced coelomocytes was detected by flow cytometry. Ajβ-actin served as the control. (**H**) The intracellular bacteria were measured in coelomocytes post siNC + *V*. *splendidus* or si-circ-1 + *V*. *splendidus* treatment. The coelomocytes were collected, lysed, diluted 1000-fold, and inoculated onto 2216E agar plates overnight, after which the colonies were counted. The data are presented as the means ± SDs; n = 3. *P < 0.05, **P < 0.01, ***P < 0.001 indicate the significant differences.

To investigate the role and underlying mechanism of circ-FGL1 in the development of SUS-diseased *A*. *japonicus*, we designed two specific circ-FGL1 siRNAs (si-circ-1 and si-circ-2). These siRNAs were transfected into coelomocytes, and the knockdown efficiency was evaluated using qRT-PCR. As depicted in Fig 2A, both si-circ-1 and si-circ-2 selectively targeted circ-FGL1 in a dose-dependent manner, with si-circ-1 showing the most effective knockdown. We also observed that the knockdown of circ-FGL1 using si-circ-1 did not affect the expression levels of AjFGL1 mRNA or protein (Fig 2B). Subsequently, RNA-seq analysis was performed on sea cucumber coelomocytes treated with circ-FGL1 siRNA (si-circ-1) and the siNC control. Compared to control coelomocytes, circ-FGL1-silenced cells revealed significant enrichment of the apoptosis pathway from the gene ontology (GO) analysis (Fig 2C). The effects of circ-FGL1 on coelomocyte apoptosis during *V*. *splendidus* challenge *in vivo* and LPS exposure *in vitro* were then investigated. Flow cytometry data showed that circ-FGL1 silencing increased the apoptosis rate of coelomocytes upon treatment with *V*. *splendidus* or LPS (Fig 2D). Consistently, the mRNA and protein expression levels of AjCytochrome c (AjCyt c) and Ajcaspase 3 indicated that circ-FGL1 silencing remarkably promoted the activation of the apoptosis pathway (Fig 2E and 2F). Furthermore, the effects of circ-FGL1 on the apoptosis pathway were confirmed by flow cytometry analysis after Ajcaspase 3 and AjCyt c knockdown (Fig 2G). Moreover, the impact of circ-FGL1 on *V*. *splendidus* invasion of coelomocytes was evaluated using a plate counting method to calculate the number of intracellular bacteria. As shown in Fig 2H, circ-FGL1 silencing significantly reduced the number of *V*. *splendidus* invading coelomocytes (Fig 2H). Overall, these results confirm that circ-FGL1 inhibits the apoptosis signaling pathway in *A*. *japonicus* in response to pathogen infection.

### circ-FGL1 directly interacts with AjMyc and promotes its degradation by ubiquitination

**Fig 3:**
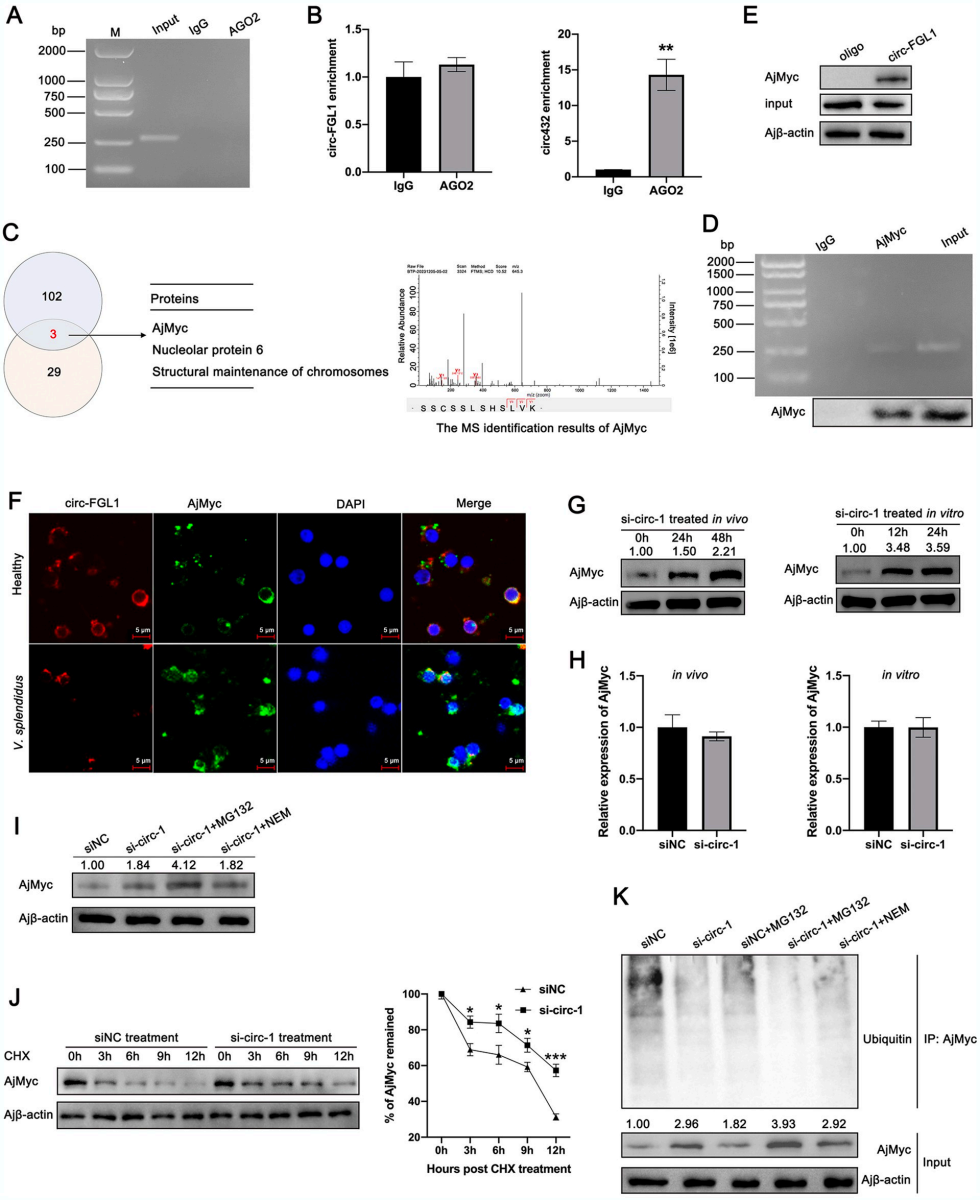
circ-FGL1 interacts with AjMyc and promotes its degradation. (**A**) circ-FGL1, which was enriched in coelomocytes by anti-AGO2 antibodies, was detected by RIP, followed by agarose gel electrophoresis analysis. Pre-immune serum served as the negative control. (**B**) circ-FGL1, which was enriched in coelomocytes by anti-AGO2 antibodies, was detected by RIP, followed by qRT-PCR analysis. circ432 served as the control. (**C**) A list of potential circ-FGL1-interacting proteins identified by RNA pull-down followed by the MS in both coelomocytes and intestines. The results of MS identification of AjMyc are shown on the right. (**D**) circ-FGL1, which was enriched by AjMyc antibodies or pre-immune serum, in coelomocytes was detected by RIP, followed by agarose gel electrophoresis analysis. The protein levels of AjMyc enriched by pre-immune serum, AjMyc antibodies, and in untreated coelomocytes are shown below. (**E**) The AjMyc protein in coelomocytes enriched with circ-FGL1 was detected by RNA pull-down assays followed by western blotting. Oligo served as the control. (**F**) The co-localization of circ-FGL1 and AjMyc in coelomocytes of healthy and *V*. *splendidus*-challenged sea cucumbers was detected by RNA FISH and immunofluorescence. circ-FGL1 (red), AjMyc (green), and nuclei (blue) were stained with Alexa Fluor 594, FITC, and DAPI, respectively. (**G**, **H**) AjMyc protein and mRNA expression levels in si-circ-1-transfected coelomocytes were measured by western blotting and qRT-PCR *in vivo* and *in vitro*. (**I**) Changes in AjMyc protein expression in si-circ-1-transfected coelomocytes post MG132 or NEM treatment were measured by western blotting. Coelomocytes transfected with siNC served as the control. (**J**) Changes in AjMyc protein expression in si-circ-1- or siNC-transfected coelomocytes post CHX treatment were measured by western blotting. Band density analysis was performed at each detection time point. (**K**) The ubiquitination levels of AjMyc in coelomocytes transfected with si-circ-1 or siNC for 24 h and then treated with MG132 or NEM for 6 h were detected by western blotting. Ajβ-actin served as the control. The data are presented as the means ± SDs; n = 3. *P < 0.05, **P < 0.01, and ***P < 0.001 indicate a significant difference.

To further investigate the mechanisms underlying the inhibition of apoptosis by circ-FGL1 during pathogen infection, we first assessed whether circ-FGL1 could be pulled down by the Argonaute 2 (AGO2) proteins [47]. We confirmed this finding using a RIP assay, which demonstrated that circ-FGL1 cannot be pulled down by the AGO2 protein (Fig 3A). Additionally, qRT-PCR revealed that AGO2 monoclonal antibodies were able to precipitate circRNA 432 (a circRNA previously known to act as a miRNA sponge in *A*. *japonicus*) [3] but not circ-FGL1 (Fig 3B), which indicated that circ-FGL1 does not function as a miRNA sponge. Furthermore, we observed that the mRNA and protein levels of AjFGL1 remained unchanged following treatment with si-circ-1 (Fig 2B). Previous research has suggested that circRNAs may play a role in the development of diseases by regulating RNA binding proteins (RBPs) [18]. To verify this hypothesis, we conducted an RNA pull-down assay to identify proteins that interact with circ-FGL1, followed by MS analysis (dataset identifier: PXD051546). Given that circ-FGL1 displayed similar depressed expression in coelomocytes and intestines post *Vibrio splendidus* challenge and LPS stimulation (Fig 1G), and intestine apoptosis was also significantly induced post *V*. *splendidus* infection [48], we therefore performed the RNA pull-down assay in these two tissues. We identified a total of 101 and 29 circ-FGL1-specific interacting proteins in coelomocytes and intestines, respectively. Among these proteins, 3 were consistently detected in both tissues: *A*. *japonicus* Myc (AjMyc), nucleolar protein 6, and structural maintenance of chromosomes (Fig 3C). AjMyc was of particular interest due to its known involvement in gene transcriptional regulation [49], and it received the highest score and showed the greatest abundance in the MS analysis (S1 Fig and S1 Data). To confirm the interaction between circ-FGL1 and AjMyc, we conducted a RIP assay followed by RT-PCR to detect circ-FGL1. The results demonstrated that circ-FGL1 can be enriched by AjMyc but not by IgG (Fig 3D). The interaction between circ-FGL1 and AjMyc was also confirmed by RNA pull-down assays (Fig 3E). Additionally, we investigated the colocalization of circ-FGL1 and AjMyc in the coelomocytes of healthy and *V*. *splendidus*-challenged *A*. *japonicus*. As shown in Fig 3F, circ-FGL1 and AjMyc were predominantly colocalized in the cytoplasm of healthy sea cucumber coelomocytes. Furthermore, after infection with *V*. *splendidus*, the expression levels of circ-FGL1 and AjMyc in coelomocytes exhibited opposite trends, with increased AjMyc nuclear translocation.

Next, we determined whether the expression of AjMyc can be regulated by circ-FGL1. Interestingly, we observed that circ-FGL1 significantly affected the protein level of AjMyc both *in vivo* and *in vitro* (Fig 3G), whereas the mRNA level of AjMyc remained unchanged when circ-FGL1 was knocked down (Fig 3H). Given these observations, we suspected that circ-FGL1 regulates AjMyc in coelomocytes through posttranscriptional mechanisms. To confirm this hypothesis, we treated control and circ-FGL1-silenced coelomocytes with the protein synthesis inhibitor CHX and the proteasome inhibitor MG132. As shown in Fig 3I, the knockdown of circ-FGL1 further increased the protein level of AjMyc, and this increase was more pronounced in the presence of MG132. However, the addition of NEM (an inhibitor of endogenous deubiquitinating enzymes) did not have the same effect. Furthermore, CHX chase assays revealed that circ-FGL1 knockdown resulted in a prolonged half-life of the AjMyc protein (Fig 3J). Finally, we conducted IP assays to assess the levels of ubiquitinated proteins in control and circ-FGL1-silenced coelomocytes. The IP results showed that AjMyc was less ubiquitinated in circ-FGL1-silenced and MG132-treated coelomocytes than in control cells treated with NEM and MG132 (Fig 3K). These results indicated that circ-FGL1 promotes AjMyc ubiquitination levels in coelomocytes post-transcriptionally.

### Identification of the binding regions between circ-FGL1 and AjMyc

**Fig 4:**
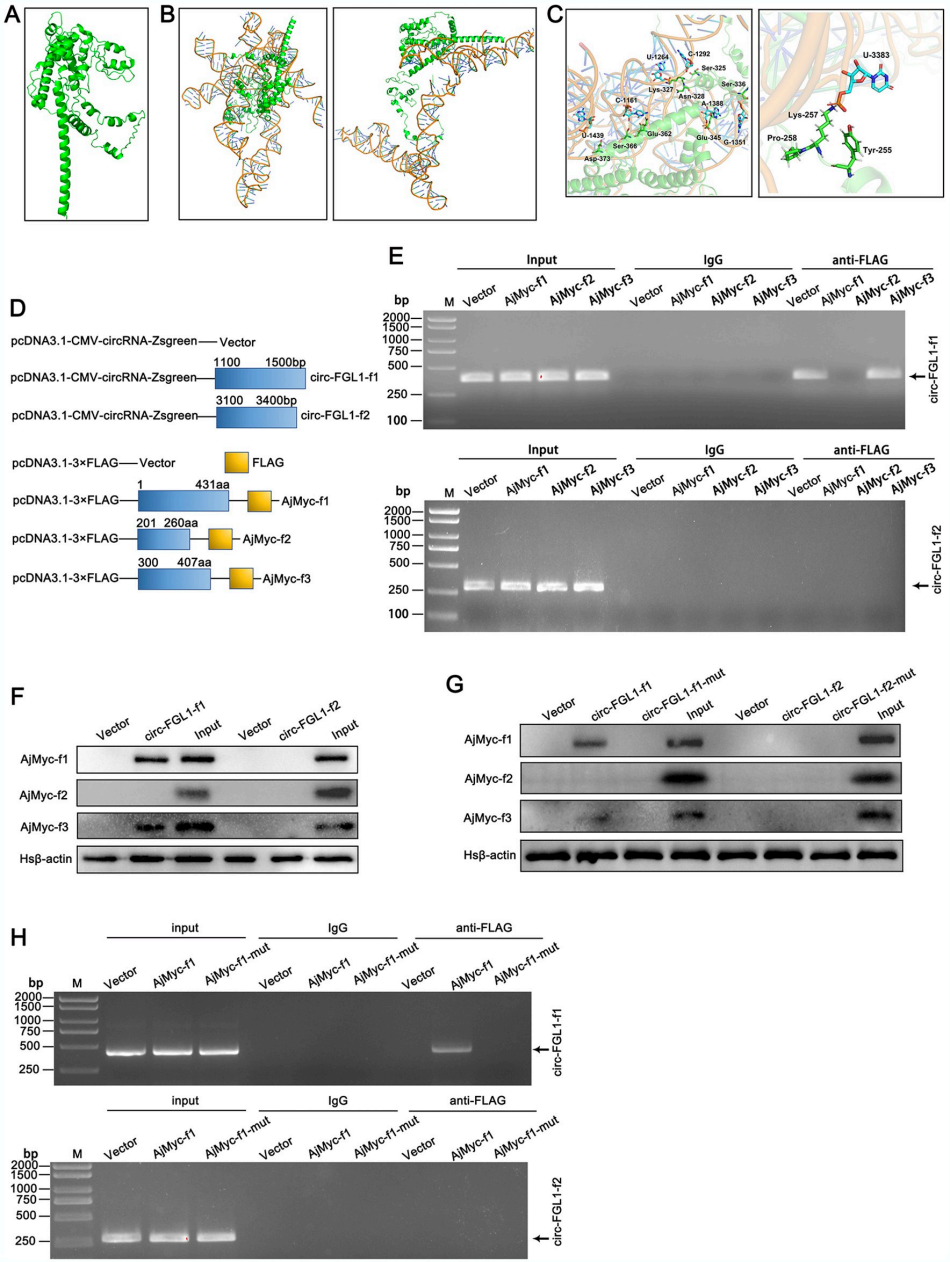
Interaction of AjMyc with circ-FGL1. (**A**) A 3-D model of AjMyc was constructed with the AlphaFold 2 program. (**B**) Graphical representation of 3-D structures of the docking models of circ-FGL1 with AjMyc. circ-FGL1 was predicted to interact with AjMyc to form a complex at two parts, circ-FGL1 (1100–1500 bp) and AjMyc (300–407 aa) on the left, and circ-FGL1 (3100–3400 bp) and AjMyc (200–257 aa) on the right. (**C**) Nucleotides or aa are involved in the interaction of circ-FGL1 (green) with AjMyc (indigo). (**D**) Schematic diagrams of the vectors used to construct different regions of circ-FGL1 and AjMyc were constructed based on their interaction regions. (**E**) RIP assays were performed with circ-FGL1-f1- (upper) or circ-FGL1-f2- (lower), and AjMyc-f1-, AjMyc-f2-, or AjMyc-f3-transfected HEK 293T cells using anti-FLAG antibodies or pre-immune serum followed by RT-PCR analysis. (**F**) RNA pull-down assays were performed with circ-FGL1-f1-, circ-FGL1-f2-, AjMyc-f1-, AjMyc-f2-, and AjMyc-f3-transfected using circ-FGL1-f1 and circ-FGL1-f2 probes followed by western blotting analysis. HEK 293T cells transfected with empty vectors served as controls. (**G**) RNA pull-down assays were performed in circ-FGL1-f1-, circ-FGL1-f2-, circ-FGL1-f1-mut-, circ-FGL1-f2-mut-, AjMyc-f1-, AjMyc-f2-, or AjMyc-f3-transfected HEK 293T cells using circ-FGL1-f1 and circ-FGL1-f2 probes followed by western blotting analysis. HEK 293T cells transfected with empty vectors served as controls. (**H**) RIP assays were performed in circ-FGL1-f1- (upper), or circ-FGL1-f2- (lower), and AjMyc-f1-, or AjMyc-f1-mut-transfected HEK 293T cells using anti-FLAG antibodies or pre-immune serum followed by RT-PCR analysis.

As the full-length structure of AjMyc in echinoderms has not been identified, we initially predicted a three-dimensional (3-D) model using the AlphaFold 2 program (Fig 4A). Then, the secondary structure of circ-FGL1 was predicted and analyzed through the formula ΔG = ΔH-TΔS, ΔG = 10089.7 kcal/mol at 37°C, ΔH = 57212.5 kcal/mol, and ΔS = 151925.8 kcal/(K·mol). T(K) indicates the absolute temperature and ΔH, ΔS, and ΔG, represent the change in enthalpy, entropy, and free energy, respectively. The tertiary structure of circ-FGL1 was then predicted and analyzed by the RNA composer software derived from its secondary structure. Next, we used two bioinformatic programs (catRAPID and NPdock) to predict the minimum binding region and binding sites of circ-FGL1 and AjMyc. The results indicated that circ-FGL1 could form a complex with AjMyc at two regions: circ-FGL1 (1100–1500 bp) and AjMyc (300–407 aa), and circ-FGL1 (3100–3400 bp) and AjMyc (200–257 aa) (Fig 4B). The necessary nucleotides of circ-FGL1 for binding to AjMyc were shown in Fig 4C.

To investigate the interaction between endogenous circ-FGL1 and AjMyc, we constructed a series of circ-FGL1 and AjMyc truncations based on their predicted binding sites, including pcDNA3.1-CMV-circRNA-Zsgreen vector constructed circ-FGL1-f1 (1100–1500 bp) and circ-FGL1-f2 (3100–3400 bp), and pcDNA3.1–3×FLAG-C vector constructed AjMyc-f1 (full length ORF, 1–431 aa), AjMyc-f2 (201–260 aa), and AjMyc-f3 (300–407 aa) (Fig 4D). HEK 293T cells were then transfected with circ-FGL1 truncations and vectors encoding various fragments of the AjMyc protein. The results of the RIP and RNA pull-down assays showed that circ-FGL1-f1 interacts with AjMyc-f3 (Fig 4E and 4F), which matched the predicted binding sites described in Fig 4D. Additionally, the expression levels of AjMyc-f1, AjMyc-f2, and AjMyc-f3 in HEK 293T cells determined by RIP assays are shown in S2 Fig. Furthermore, we mutated the predicted binding sites in circ-FGL1-f1 (1100–1500 bp) and AjMyc-f3 (300–407 aa) to detect whether these mutants still interact with circ-FGL1 and AjMyc, respectively (S2 Data). The results showed that the wild-type of circ-FGL1 or AjMyc, interacted with AjMyc or circ-FGL1, respectively, whereas the mutants did not see (Fig 4G and 4H). The expression of AjMyc-f1 and its mutant, AjMyc-f1-mut, in HEK 293T cells is shown in S3 Fig.

### AjOTUB1 interacts with AjMyc, enhances its protein stability, and deubiquitinates AjMyc at the same region as circ-FGL1

**Fig 5:**
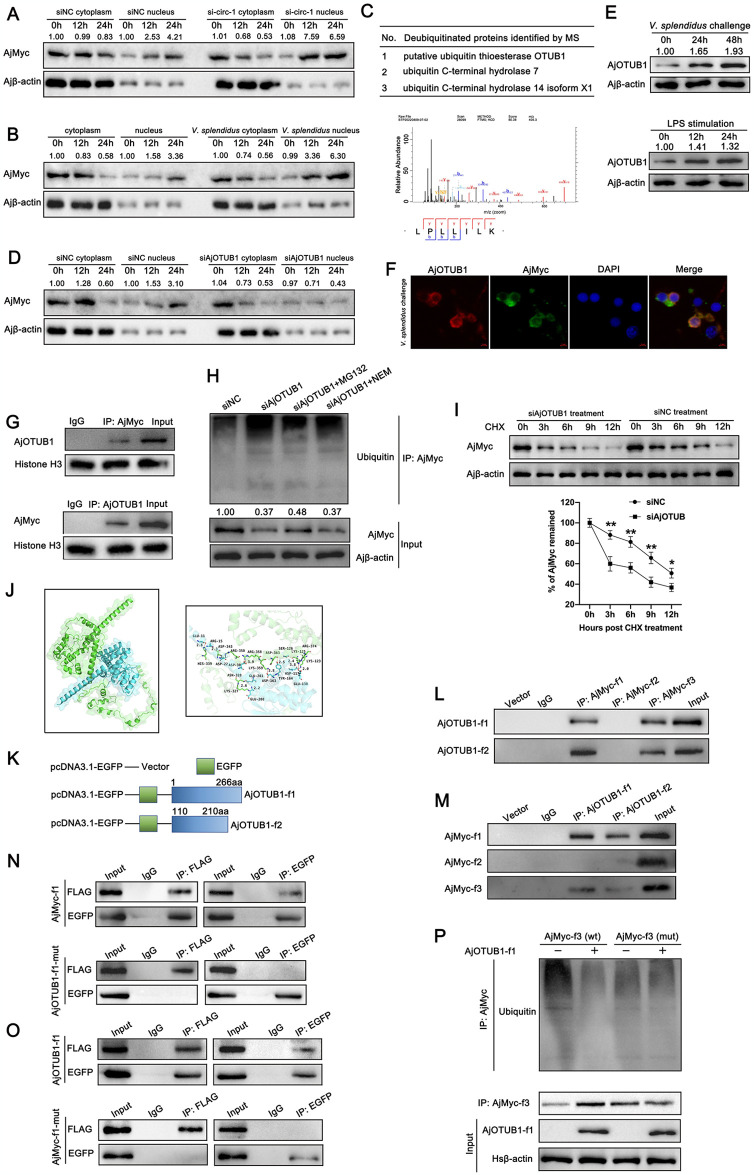
AjOTUB1 interacts with AjMyc and deubiquitinates AjMyc at the same region as circ-FGL1. (**A**) AjMyc nuclear translocation in si-circ-1- or siNC-transfected coelomocytes was investigated at 0, 12, and 24 h by western blotting. (**B**) AjMyc nuclear translocation in *V*. *splendidus*-challenged or untreated *A*. *japonicus* coelomocytes was investigated at 0, 12, and 24 h by western blotting. (**C**) A list of potential AjMyc-interacting deubiquitinases identified by immunoprecipitation followed by MS analysis in coelomocytes. The results of the MS identification of AjOTUB1 are shown below. (**D**) AjMyc nuclear translocation in siAjOTUB1 or siNC transfected-coelomocytes at 0, 12, and 24 h was investigated by western blotting. (**E**) AjOTUB1 protein expression in coelomocytes at 0, 24, and 48 h post *V*. *splendidus* challenge or LPS stimulation was detected by western blotting. (**F**) The co-localization of AjMyc and AjOTUB1 in *V*. *splendidus* challenged sea cucumber coelomocytes was detected by immunofluorescence. AjMyc (green), AjOTUB1 (red), and nuclei (blue) were stained with FITC, Alexa Fluor 594, and DAPI, respectively. Bar: 2 μM. (**G**) co-IP assays were performed to detect the interaction between AjMyc and AjOTUB1 in the nuclei of sea cucumbers challenged with *V*. *splendidus* by western blotting. Histone H3 served as the internal reference. (**H**) The level of ubiquitinated AjMyc in coelomocytes transfected with siAjOTUB1 for 24 h and then treated with MG132 or NEM for 6 h was detected by western blotting. Coelomocytes transfected with siNC served as the control. (**I**) Changes in AjMyc protein expression in siAjOTUB1- or siNC-transfected coelomocytes post CHX treatment was measured by western blotting. Band density analysis was performed at each detection time point. (**J**) Graphical representation of 3-D structures of the docking models of AjMyc and AjOTUB1, and the amino acids involved in the interaction between AjMyc (green) and AjOTUB1 (indigo). (**K**) Schematic diagrams of the AjOTUB1 vectors constructed based on the regions where AjOTUB1 interacts with AjMyc. (**L**) co-IP assays were performed in AjOTUB1-f1- or AjOTUB1-f2-, and AjMyc-f1-, AjMyc-f2-, or AjMyc-f3-transfected HEK 293T cells using anti-FLAG antibodies or pre-immune serum followed by western blotting detection with anti-GFP antibodies. (**M**) co-IP assays were performed in AjMyc-f1-, AjMyc-f2-, or AjMyc-f3-, and AjOTUB1-f1- or AjOTUB1-f2-transfected HEK 293T cells using anti-GFP antibodies or pre-immune serum followed by western blotting detection with anti-FLAG antibodies. (**N**, **O**) co-IP assays were performed in AjOTUB1-f1-, AjOTUB1-f1-mut-, AjMyc-f1-, and AjMyc-f1-mut-transfected HEK 293T followed by western blotting detection with anti-FLAG or anti-GFP antibodies. (**P**) IP and western blotting were performed to detect the ubiquitination levels of both EGFP-tagged AjOTUB1 and FLAG-tagged AjMyc (wt or mut)-transfected HEK 293T cells. The levels of AjMyc in the cytoplasm or nucleus of siNC-transfected or untreated-coelomocytes at 0 h served as the control, and the levels of AjMyc nuclear translocation were comparatively analyzed at each time point between siNC- and si-circ-1-treated cytoplasm or nucleus, untreated- and *V*. *splendidus*-challenged cytoplasm or nucleus, and siNC- and siAjOTUB1-treated cytoplasm or nucleus. Ajβ-actin and Hsβ-actin served as the controls. The data are presented as the means ± SDs; n = 3. *P < 0.05 and **P < 0.01 indicate a significant difference.

It has been reported that Myc can regulate up to 15% of gene expression [50]. In this study, we observed significant downregulation of circ-FGL1 in *V*. *splendidus*-challenged or LPS-stimulated coelomocytes (Fig 1G and 1H), and found that circ-FGL1 regulates AjMyc levels in coelomocytes post-transcriptionally (Fig 3G and 3H). Given that Myc functions as a transcription factor, we thus detected whether circ-FGL1 could induce AjMyc nuclear translocation. The results revealed that circ-FGL1 silencing markedly promoted AjMyc nuclear translocation (Fig 5A). Previous evidence has shown that nuclear Myc is readily degraded [51,52]. In the present study, high levels of AjMyc translocated to the nucleus following circ-FGL1 knockdown or *V*. *splendidus* infection, but we did not observe a decrease in the total protein level of AjMyc under these conditions (Fig 5A and 5B). The transcription level of AjMyc nuclear translocation in coelomocytes after *V*. *splendidus* challenge was similar to that of AjMyc protein expression under the same conditions (S4 Fig). This finding suggested the involvement of another protein in preventing AjMyc degradation [53]. To this aim, we conducted the co-IP assays followed by MS to identify the specific deubiquitinating enzyme that prevents AjMyc protein degradation. A total of 3 deubiquitinating enzymes were identified to interact with AjMyc in sea cucumber coelomocytes (dataset identifier: PXD051548) (Fig 5C). Then, we detected the expression levels of AjMyc in coelomocytes treated with specific siRNAs targeting three deubiquitinating enzymes (siAjOTUB1, siAjUCH7, and siAjUCH14). The results showed no change in the mRNA levels of AjMyc following the silencing of the three deubiquitinating enzymes (S5 Fig). On the other hand, the protein levels of AjMyc, but not those of the other two targeted deubiquitinating enzymes, were significantly reduced in coelomocytes after the knockdown of *A*. *japonicus* OTU domain-containing ubiquitin aldehyde-binding protein 1 (AjOTUB1) (S6 Fig). Furthermore, silencing AjOTUB1 markedly decreased AjMyc nuclear translocation in sea cucumber coelomocytes (Fig 5D).

OTUB1, a member of the OTU family, plays a role in mediating protein deubiquitination and has been reported to regulate immune functions in various human diseases [53,54]. However, the mechanisms underlying the association between ncRNAs and the degradation of target proteins mediated by OTUB1 remain unclear. In this study, we successfully cloned the full-length cDNA of AjOTUB1, which consists of a 166 bp 5′-UTR, a 3′-UTR of 3165 bp, and an 801 bp ORF with a classic peptidase_C65 domain (GenBank accession no. PP690938) (S7 Fig). Multiple sequence alignment and phylogenetic analysis (S8 and S9 Figs), and tertiary structure analysis (S10 Fig) all support that AjOTUB1 belongs to the OTUB1 family. Additionally, we observed a significant increase in AjOTUB1 protein levels in coelomocytes following *V*. *splendidus* challenge or LPS stimulation (Fig 5E). Subsequently, we investigated whether AjOTUB1 directly interacts with AjMyc. Immunofluorescence analysis revealed that AjMyc and AjOTUB1 were predominantly colocalized in the nuclei of coelomocytes upon *V*. *splendidus* challenge (Fig 5F). Co-IP assays further confirmed the specific interaction between AjMyc and AjOTUB1 in the nucleus (Fig 5G). Previous studies have demonstrated that OTUB1, which functions as a deubiquitinase, can deubiquitinate various substrates to regulate protein stability [53]. To explore whether AjOTUB1 regulates AjMyc protein stability through the proteasome pathway, we treated coelomocytes with MG132 and CHX. The results indicated that MG132 promoted AjOTUB1-mediated stability of the AjMyc protein, whereas CHX increased the half-life of the AjMyc protein in coelomocytes (Fig 5H and 5I), indicating that AjOTUB1 enhances AjMyc protein stability through the ubiquitin-proteasome pathway.

The AphaFold2 program was performed to predict the 3-D protein structure of AjOTUB1. Subsequently, potential binding partners between AjOTUB1 and AjMyc were identified using the Zdock server and PyMOL open-source software. The results revealed the ability of these two proteins to form a complex at the 300–400 aa region of AjMyc and the 110–210 aa of AjOTUB1. As shown in Fig 5J, the amino acids necessary for AjOTUB1 binding to AjMyc are also shown. To further investigate the AjMyc regions that interact with AjOTUB1 *in vitro*, a series of AjOTUB1 and AjMyc truncation mutants were generated based on their predicted binding sites. These included the pcDNA3.1-EGFP vector, which contained the full-length ORF of AjOTUB1 (AjOTUB1-f1, 1–266 aa), AjOTUB1 truncations (AjOTUB1-f2, 110–210 aa), and various fragments of the AjMyc protein as described earlier (AjMyc-f1 (1–431 aa), AjMyc-f2 (201–260 aa), and AjMyc-f3 (300–407 aa)) (Fig 5K). Our results of co-IP assays confirmed that both AjMyc-f1 (full-length) and AjMyc-f3 (300–407 aa) interact with AjOTUB1-f1 and AjOTUB1-f2 (Fig 5L and 5M). Furthermore, we mutated the predicted binding sites in AjMyc-f3 regions (S2 Data) and the interaction between AjMyc and AjOTUB1 was investigated. We found that AjMyc-wt, but not AjMyc-mut, interacted with AjOTUB1 in coelomocytes (Fig 5N and 5O). In addition, as expected, AjOTUB1 failed to maintain the protein stability of the AjMyc-f3 mutant (Fig 5P). Collectively, we concluded that AjOTUB1 interacts with AjMyc, enhances its protein stability, and deubiquitinates AjMyc in the same region as circ-FGL1.

### High levels of circ-FGL1 preferentially bind to the AjMyc protein in competition with AjOTUB1 and inhibit AjOTUB1-mediated AjMyc deubiquitination

**Fig 6:**
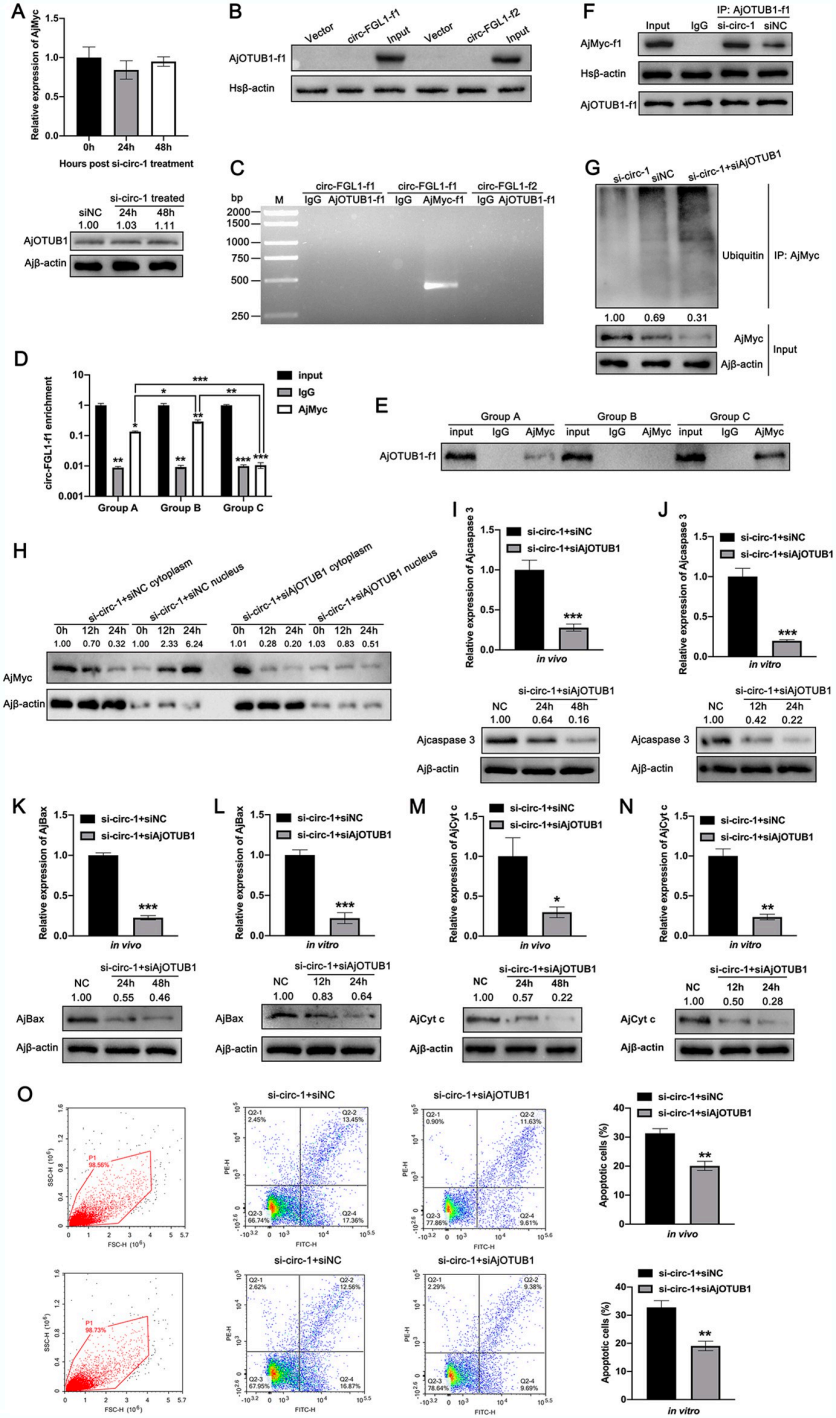
AjOTUB1 protects against circ-FGL1-mediated AjMyc protein degradation. (**A**) AjOTUB1 mRNA and protein expression levels in si-circ-1-transfected coelomocytes were detected by qRT-PCR and western blotting, respectively. (**B**) RNA pull-down assays were performed in AjOTUB1-f1-, and circ-FGL1-f1- or circ-FGL1-f2-, or empty vector-transfected HEK 293T cells using circ-FGL1-f1 and circ-FGL1-f2 probes followed by western blotting detection with anti-GFP antibodies. (**C**) RIP assays were performed in AjMyc-f1- and circ-FGL1-f1-, circ-FGL1-f2-, or AjOTUB1-f1-transfected HEK 293T cells using anti-FLAG antibodies, anti-GFP antibodies or pre-immune serum followed by RT-PCR analysis. (**D**) qRT-PCR detection of AjMyc-enriched circ-FGL1-f1 in the circ-FGL1-f1 + rAjMyc + rAjOTUB1, circ-FGL1-f1 + rAjMyc + rAjOTUB1-mut, and circ-FGL1-f1-mut + rAjMyc + rAjOTUB1 groups using anti-FLAG antibodies. (**E**) co-IP analysis of AjOTUB1 immunoprecipitated by FLAG-tagged AjMyc in the circ-FGL1-f1 + rAjMyc + rAjOTUB1, circ-FGL1-f1 + rAjMyc + rAjOTUB1-mut, and circ-FGL1-f1-mut + rAjMyc + rAjOTUB1 groups using anti-GFP antibodies. (**F**) co-IP assays were conducted in si-circ-1- or siNC-transfected HEK 293T cells using anti-GFP antibodies or pre-immune serum followed by western blotting detection with anti-FLAG antibodies. (**G**) The level of ubiquitinated AjMyc in si-circ-1-transfected coelomocytes after si-AjOTUB1 treatment was detected by western blotting with anti-AjMyc antibodies. Ajβ-actin served as the control. (**H**) AjMyc nuclear translocation in si-circ-1 transfected-coelomocytes post siAjOTUB1 treatment was investigated at 0, 12, and 24 h by western blotting. The levels of AjMyc in the cytoplasm or nucleus of siNC + si-circ-1-transfected coelomocytes at 0 h served as the control, and the levels of AjMyc nuclear translocation were comparatively analyzed at each time point in the siNC + si-circ-1- and si-circ-1 +siAjOTUB1-treated cytoplasm or nucleus. (**I, J, K, L, M, N**) The mRNA and protein expression levels of Ajcaspase 3 (I, J), AjBax (K, L), and AjCyt c (M, N) in si-circ-1-transfected sea cucumber coelomocytes or cultured primary coelomocytes post siAjOTUB1 or siNC treatment were detected by qRT-PCR and western blotting, respectively. (**O**) The apoptosis levels in si-circ-1-transfected sea cucumber coelomocytes or cultured primary coelomocytes post siAjOTUB1 or siNC treatment were investigated by flow cytometry. The data are presented as the means ± SDs; n = 3. *P < 0.05, **P < 0.01, and ***P < 0.001 indicate the significant differences.

Given that circ-FGL1 and AjOTUB1 bind to the same region of the AjMyc protein and exhibit opposite expression patterns under *V*. *splendidus* challenge, we hypothesized that AjOTUB1 may compete with circ-FGL1 for binding to AjMyc. To validate this hypothesis, we first assessed the expression levels of AjOTUB1 post circ-FGL1 treatment. The results showed that the mRNA and protein expression levels of AjOTUB1 were not affected in circ-FGL1-silenced coelomocytes (Fig 6A). Furthermore, RNA pull-down assays did not detect any direct interaction between circ-FGL1 and AjOTUB1 (Fig 6B). RIP assays confirmed that circ-FGL1 interacts with AjMyc but not AjOTUB1 (Fig 6C). To further investigate binding preferences, we immobilized the FLAG-tagged AjMyc-f1 protein on FLAG resin, and then purified circ-FGL1-f1 plasmids and EGFP-tagged AjOTUB1-f1 protein (Group A), purified circ-FGL1-f1 plasmids and EGFP-tagged AjOTUB1-f1 mutant protein (Group B), circ-FGL1-f1-mut plasmids and EGFP-tagged AjOTUB1-f1 protein (Group C) were added to the FLAG-AjMyc-conjugated resin, respectively. We compared the expression levels of circ-FGL1 and AjOTUB1 in combination with AjMyc among the three groups. The specific treatment strategy is outlined in S2 Table. qPCR analysis revealed that the levels of circ-FGL1-f1 immunoprecipitated by AjMyc were significantly greater in Group B than in Group A, but the levels were not investigated in Group C under the same conditions (Fig 6D). Additionally, co-IP revealed that the protein levels of AjOTUB1 immunoprecipitated by AjMyc were much greater in Group C than in Group A, whereas no AjOTUB1 protein was detected in Group B (Fig 6E). These results indicated that circ-FGL1 may preferentially bind to the AjMyc protein in competition with AjOTUB1. More importantly, the results of co-IP assays showed that the silencing of circ-FGL1 in coelomocytes promoted the interaction between AjOTUB1 and AjMyc in the nucleus (Fig 6F). Ubiquitination assays also revealed that AjOTUB1 knockdown significantly increased the level of ubiquitinated AjMyc following circ-FGL1 depletion(Fig 6G). Moreover, compared to those in si-circ-1- and siNC-treated coelomocytes, we observed a significant decrease in AjMyc nuclear translocation in si-circ-1- and siAjOTUB1-treated sea cucumber coelomocytes (Fig 6H). Additionally, we detected the mRNA and protein expression levels of AjBax, Ajcaspase 3 and AjCyt c in coelomocytes post si-circ-1 and siAjOTUB1 treatment both *in vivo* and *in vitro*. The results showed that siAjOTUB1 significantly decreased the mRNA and protein expression levels of these three genes in si-circ-1-treated coelomocytes *in vivo* and *in vitro* (Fig 6I, 6J, 6K, 6L, 6M and 6N). As expected, we also found that the apoptosis levels in siAjOTUB1 and si-circ-1-treated coelomocytes were much decreased *in vivo* and *in vitro*, compared to the coelomocytes treated with siNC and si-circ-1 (Fig 6O). In addition, the mRNA and protein levels of AjBax, Ajcaspase 3, and AjCyt c were markedly decreased in *V*. *splendidus*-challenged sea cucumber coelomocytes and LPS-exposed cultured primary coelomocytes following siAjOTUB1 treatment (S11 Fig). Taken together, these results indicate that circ-FGL1 has a greater affinity for AjMyc in competition with AjOTUB1 and that highly expressed AjOTUB1 preferentially binds to the AjMyc protein, preventing its degradation during *V*. *splendidus* infection.

### AjMyc mediates the effects of circ-FGL1 on coelomocyte apoptosis

**Fig 7:**
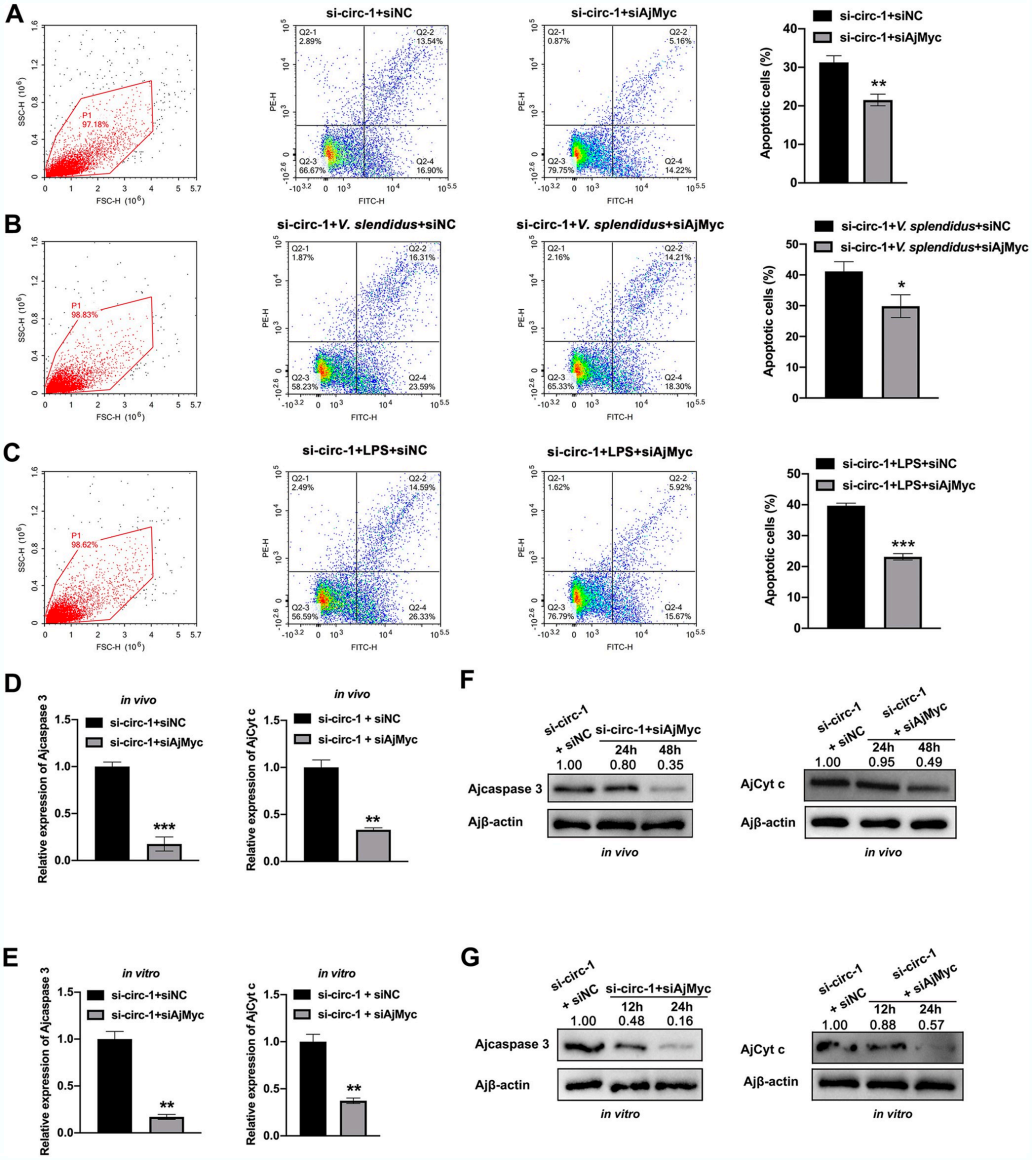
circ-FGL1 regulates coelomocyte apoptosis via AjMyc. (**A**) AjMyc functions in the apoptosis of si-circ-1-treated coelomocytes were measured by flow cytometry. Coelomocytes treated with si-circ-1 and siNC served as the controls. (**B**) The levels of coelomocyte apoptosis levels in si-circ-1 and *V*. *splendidus*-treated sea cucumbers treated with siAjMyc or siNC were measured by flow cytometry. (**C**) The apoptosis levels in si-circ-1 and LPS-treated cultured primary coelomocytes treated with siAjMyc or siNC were measured by flow cytometry. (**D**, **F**) The mRNA and protein expression levels of Ajcaspase 3 and AjCyt c in si-circ-1-treated sea cucumber coelomocytes and in those treated with siAjMyc or siNC were measured by qRT-PCR and western blotting, respectively. (**E**, **G**) The mRNA and protein expression levels of Ajcaspase 3 and AjCyt c in si-circ-1-treated cultured primary coelomocytes and in those treated with siAjMyc or siNC were measured by qRT-PCR and western blotting, respectively. Ajβ-actin served as the control. The data are presented as the means ± SDs; n = 3. *P < 0.05, **P < 0.01, ***P < 0.001 indicates the significant difference.

Our previous study demonstrated the positive regulation of coelomocyte apoptosis in sea cucumbers by AjMyc [55]. Thus, we sought to investigate the essential role of AjMyc in the function of circ-FGL1 in coelomocyte apoptosis. Flow cytometry assays revealed that the knockdown of AjMyc expression negated the increase in coelomocyte apoptosis induced by circ-FGL1 silencing (Fig 7A). The crucial involvement of AjMyc in circ-FGL1-mediated apoptosis of coelomocytes challenged with *V*. *splendidus* or stimulated with LPS was further confirmed through *in vivo* and *in vitro* flow cytometry (Fig 7B and 7C). To further explore whether AjMyc mediates the function of circ-FGL1 in the pro-apoptotic signaling pathway, the transcription levels of Ajcaspase 3 and AjCyt c were investigated. The results revealed that the mRNA levels of these two genes were significantly reduced, confirming the role of AjMyc in mediating the proapoptotic function of circ-FGL1 (Fig 7D and 7E). Correspondingly, western blotting analysis of these apoptotic markers under the same conditions yielded similar results (Fig 7F and 7G). Accordingly, these results indicated that AjMyc mediates the effects of circ-FGL1 on apoptosis upon pathogen infection.

### circ-FGL1 regulates *V*. *splendidus*-induced coelomocyte apoptosis through the AjMyc-AjBax axis

**Fig 8:**
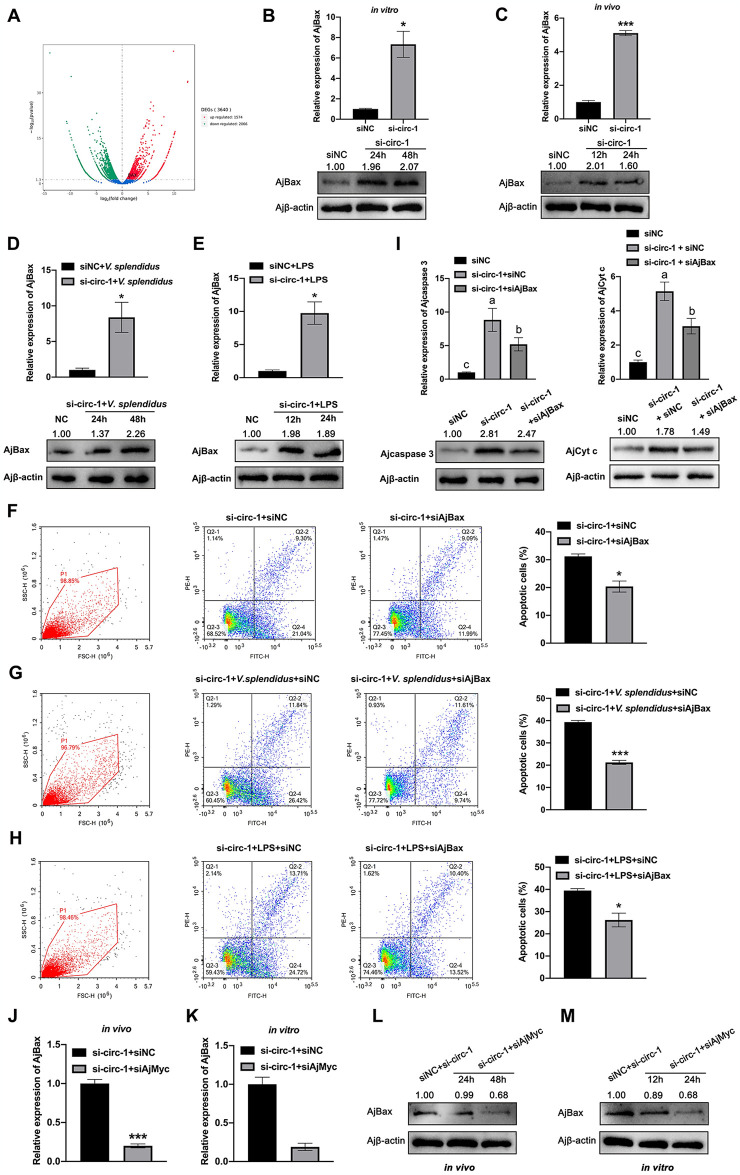
circ-FGL1 mediates coelomocyte apoptosis via the AjMyc-AjBax axis. (**A**) Heatmap detailing the differentially expressed genes derived from RNA-seq in coelomocytes post-treated by si-circ-1 and the corresponding control siNC. The differentially expressed gene, AjBax, is marked in black. (**B**, **C**) AjBax mRNA and protein expression levels in si-circ-1- or siNC-treated *A*. *japonicus* coelomocytes or cultured primary coelomocytes were measured by qRT-PCR and western blotting, respectively. (**D**, **E**) AjBax mRNA and protein expression levels in si-circ-1 and *V*. *splendidus*- or LPS-treated coelomocytes were investigated by qRT-PCR and western blotting, respectively. (**F**) AjBax functions in the apoptosis of si-circ-1-treated coelomocytes were measured by flow cytometry. (**G**) Coelomocyte apoptosis levels post si-circ-1 and *V*. *splendidus* challenge followed by transfection with siAjBax or siNC were detected by flow cytometry. (**H**) Coelomocyte apoptosis levels post si-circ-1 and LPS stimulation followed by transfection with siAjBax or siNC were detected by flow cytometry. (**I**) The mRNA and protein expression levels of Ajcaspase 3 and AjCyt c in si-circ-1-transfected coelomocytes treated with siAjBax or siNC were detected by qRT-PCR and western blotting, respectively. (**J**, **K**) AjBax mRNA expression levels in si-circ-1-transfected coelomocytes treated with siAjMyc or siNC were detected by qRT-PCR *in vivo* and *in vitro*. (**L**, **M**) AjBax protein expression levels in si-circ-1-transfected coelomocytes treated with siAjMyc or siNC were detected by western blotting *in vivo* and *in vitro*. Ajβ-actin served as the control. The data are presented as the means ± SDs; n = 3. *P < 0.05, and ***P < 0.001 indicate the significant differences.

To further elucidate the mechanism underlying circ-FGL1-mediated coelomocyte apoptosis, we performed RNA-seq analysis and identified several apoptosis signaling pathways that were altered upon circ-FGL1 knockdown, as depicted above in Fig 2C. Among the differentially expressed genes identified through RNA-seq, *A*. *japonicus* Bax (AjBax), was notably upregulated (Fig 8A), is a famous known pro-apoptotic regulator and previous studies have demonstrated its direct targeting by AjMyc which promotes AjBax promoter activity and mediates coelomocyte apoptosis [30]. Based on these findings, we hypothesized that AjBax could act as an effector in the regulation of coelomocyte apoptosis by circ-FGL1. To test this hypothesis, we investigated whether AjBax expression is also regulated by circ-FGL1 and whether it mediates its antiapoptotic function in coelomocytes. Subsequently, we evaluated the mRNA and protein levels of AjBax in coelomocytes treated with circ-FGL1 siRNA. Interestingly, our results demonstrated a significant increase in both AjBax mRNA and protein levels following circ-FGL1 knockdown both *in vivo* and *in vitro* (Fig 8B and 8C). Similar observations were made when coelomocytes were treated with circ-FGL1 siRNA in combination with *V*. *splendidus* challenge or LPS stimulation (Fig 8D and 8E). Additionally, we performed knockdown experiments targeting AjBax in circ-FGL1-silenced coelomocytes and discovered that AjBax could reverse the effects of circ-FGL1 knockdown, as indicated by changes in the levels of apoptosis (Fig 8F, 8G and 8H) and the mRNA and protein expression levels of the apoptosis marker gene (Ajcaspase 3 and AjCyt c) (Fig 8I). Furthermore, we examined the mRNA and protein expression levels of AjBax in the coelomocytes of si-circ-1- and siAjMyc-treated sea cucumbers both *in vivo* and *in vitro* and found a significant decrease compared to those in the si-circ-1- and siNC-treated sea cucumber coelomocytes (Fig 8J, 8K, 8L and 8M). Thus, we conclusively showed that the AjMyc-AjBax axis serves as a crucial downstream effector of circ-FGL1 in the regulation of apoptosis in *A*. *japonicus*.

## Discussion

Over the past decade, the functions and underlying mechanisms of circRNAs have gradually been clarified. circRNAs display outstanding roles in the occurrence and development of vertebrate and invertebrate diseases. Our previous studies reported the involvement of several endogenous circRNAs in the regulation of SUS development by acting as endogenous competitive RNA (ceRNA) in *A*. *japonicus*. For instance, circ432 enhances coelomocyte phagocytosis by regulating the miR-2008-ELMO1 axis in SUS-diseased *A*. *japonicus* [3]. Similarly, circRNA1149 is involved in the regulation of *V*. *splendidus*-induced sea cucumber coelomocyte apoptosis by acting as miR-92a sponge and mediating AjBax expression [6]. Additionally, circMETTL7A enhances Toll pathway-mediated innate immunity and antibacterial activity by reducing miR-137 function in *V*. *splendidus*-challenged *A*. *japonicus* [19]. However, a limited number of studies have focused on the specific biological functions and molecular mechanisms of circRNAs in aquatic animals. Furthermore, no reports on the interaction between circRNAs and deubiquitinase in competing for transcription factor-mediated apoptosis, a crucial process in maintaining immune defense and homeostasis in echinoderms have been published. Due to their unique covalent closed-loop structure, circRNAs have a variety of regulatory mechanisms. Consequently, there is a pressing need for further specific research to establish the function and molecular mechanisms of circRNAs.

There is ample evidence supporting the role of circRNAs as regulators of signaling pathways in important cellular processes and gene expression [56]. circRNAs perform various molecular functions to achieve their cellular effects, such as binding to RNA, influencing gene transcription, and modulating protein translation in cytoplasmic or nuclear complexes [57–60]. In this study, we investigated the significant downregulation of circ-FGL1 in various sea cucumber tissues, including coelomocytes, after challenge with *V*. *splendidus* or exposure to LPS. We further investigated that circ-FGL1-silenced coelomocytes revealed significant enrichment of the apoptosis pathway according to GO analysis. This silencing also promoted the mRNA and protein expression of Ajcaspase 3, AjCyt c, and AjBax in coelomocytes but decreased the number of *V*. *splendidus* invading coelomocytes. Given the pivotal role of apoptosis in immune homeostasis during pathogen invasion, we concluded that circ-FGL1 suppressed SUS progression during *V*. *splendidus* infection in *A*. *japonicus*. Furthermore, we confirmed that the interaction between circ-FGL1 and AjMyc, as knocking down circ-FGL1 promoted AjMyc protein expression and nuclear translocation. We previously confirmed that AjMyc directly binds to the AjBax promoter to regulate AjBax expression and mediate coelomocyte apoptosis in *A*. *japonicus* [30]. In this work, we further elucidated that circ-FGL1 regulates coelomocyte apoptosis through the AjMyc-AjBax axis in *V*. *splendidus*-challenged *A*. *japonicus*. Myc is a well-recognized protooncogene involved in various cancers. It functions as a transcription factor closely associated with cell proliferation, apoptosis, and cell cycle regulation [61]. It has also been reported to play a critical role in pathogen invasion [62]. Our previous study revealed that *V*. *splendidus*-challenged sea cucumber coelomocytes exhibited higher AjMyc protein levels, indicating important Myc regulatory mechanisms at the protein level [55]. Normally, Myc is primarily distributed in the cytoplasm, but translocates to the nucleus post signal stimulation, and is readily degraded [50,63]. In this study, higher levels of AjMyc were translocalized to the nucleus post *V*. *splendidus* challenge or circ-FGL1 knockdown, while the overall level of AjMyc did not significantly change. This finding suggests the presence of another protein that prevents the degradation of AjMyc. Han et al., [53] recently discovered that the deubiquitinase OTUB1 can inhibit Myc degradation through ubiquitination. Our revealed that circ-FGL1 and AjOTUB1 bind to the same region of the AjMyc protein, and that AjOTUB1 and AjMyc co-localized in the cytoplasm and nucleus. The expression patterns of circ-FGL1 and AjOTUB1 were opposite during *V*. *splendidus* challenge. Additionally, we also showed that circ-FGL1 preferentially binds to the AjMyc protein, competing with AjOTUB1. Therefore, we hypothesize that AjOTUB1 may prevent the degradation of AjMyc during *V*. *splendidus* infection or circ-FGL1 knockdown. Our results indicate that AjOTUB1 interacts with AjMyc but not with circ-FGL1 to prevent circ-FGL1-induced AjMyc degradation.

Ubiquitination plays a pivotal role in regulating protein degradation and is involved in various physiological and pathological processes of numerous diseases [64]. Research has highlighted the abnormal expression of Myc, a transcription factor that can control up to 15% of gene expression, in approximately 70% of human cancers [50,65]. In breast cancer, the interaction between Myc and deubiquitinases (USP28 and USP22) reinforces protein stability and contributes significantly to cell proliferation [66,67]. Additionally, USP37 has been reported to promote the Warburg effect in cancer cells and the proliferation of lung cancer cells by deubiquitinating Myc [68]. USP16 has been identified as a novel regulator of Myc stability, promoting the growth of prostate cancer cells with high tolerance [69]. However, only one article has investigated the influence of the OTU subfamily of deubiquitinases on Myc protein stability in human breast cancer [53]. In this work, we confirmed the transcription factor AjMyc is a novel substrate of the deubiquitinase AjOTUB1. Our data confirmed that AjOTUB1 directly interacts with AjMyc protein in the 300–407 amino acid region, and stabilizes AjOTUB1 through the ubiquitin-proteasome pathway. Furthermore, we demonstrated that AjOTUB1 governs coelomocyte apoptosis by modulating AjMyc-mediated AjBax expression following *V*. *splendidus* infection. Therefore, our results bridge the gap in understanding gene transcription and stability regulation mediated by OTUB1, emphasizing the significance of investigating the role and mechanism of OTUB1 during the development and progression of SUS in *A*. *japonicus*.

The pro-apoptotic member Bax, known for its crucial involvement in cell survival through the programmed cell death process [70]. In this study, we found the regulation of AjBax by circ-FGL1 and investigated whether circ-FGL1 modulates coelomocyte apoptosis via AjBax in *A*. *japonicus*. Previous studies have established that the Bax protein predominantly resides in the cytoplasm, and upon apoptosis signal stimulation, undergoes rapid activation, translocation, and insertion into the mitochondrial outer membrane. This disrupts mitochondrial membrane integrity, triggers the release of Cyt c, activates caspase 3, and ultimately executes a pro-apoptotic role [71,72]. Our investigation employed qRT-PCR, western blotting, and flow cytometry analysis to confirm that both the mRNA and protein levels of AjBax, Ajcaspase 3, and AjCyt c, as well as coelomocyte apoptosis, were significantly higher in circ-FGL1-knockdown coelomocytes than in control cells. Furthermore, compared with those in circ-FGL1-knockdown coelomocytes, the mRNA and protein levels of these two apoptotic marker genes and the level of apoptosis were markedly lower in both circ-FGL1-knockdown and AjBax-knockdown coelomocytes. These results indicate that circ-FGL1 regulates the apoptosis pathway through the AjMyc-AjBax axis.

## Conclusion

**Fig 9:**
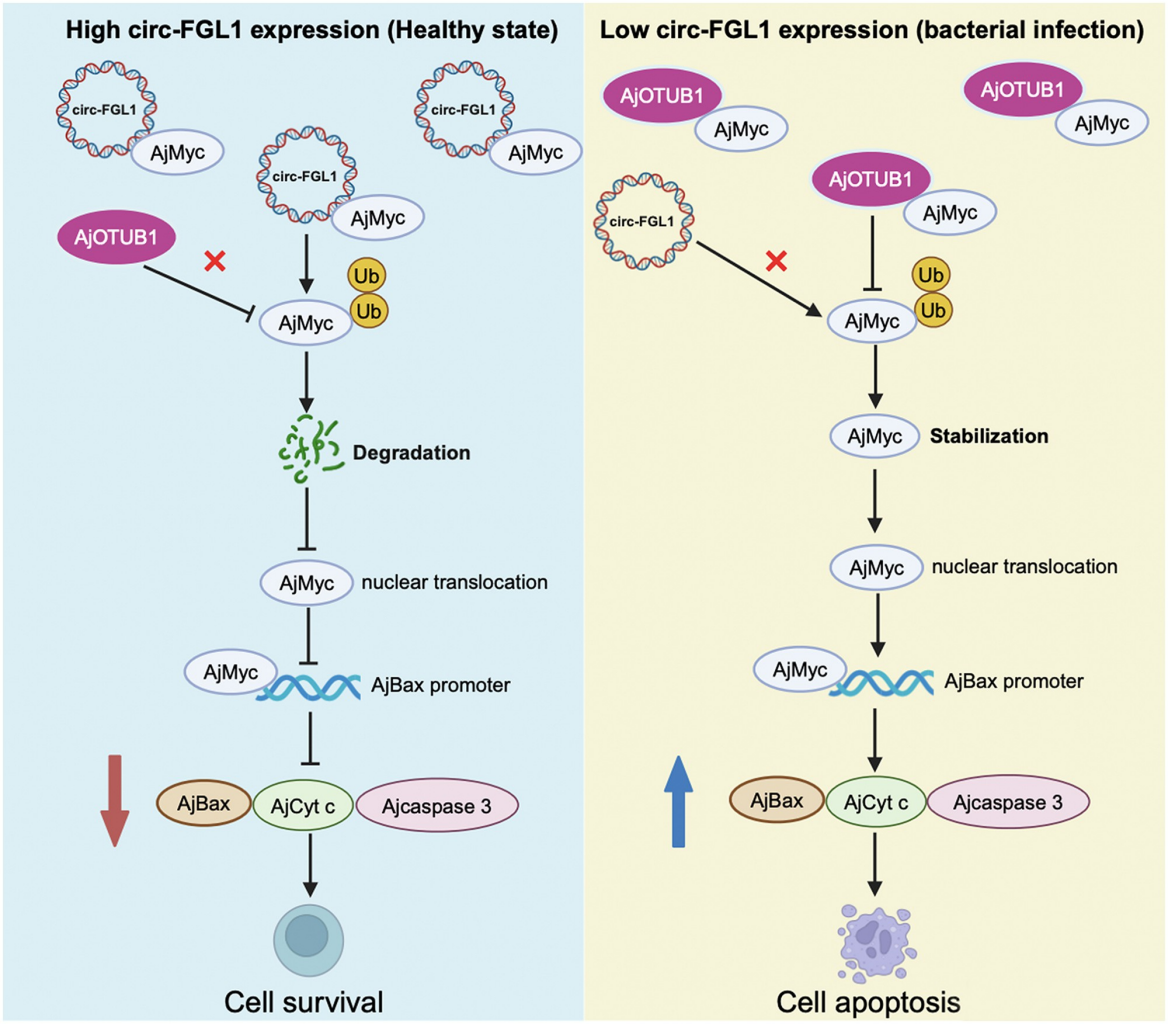
Schematic illustration of the circFGL1/AjOTUB1/AjMyc/ AjBax axis that regulates coelomocyte apoptosis in *A*. *japonicus* in response to *V*. *splendidus* infection. circ-FGL1 and AjOTUB1 were significantly downregulated and upregulated, respectively, in sea cucumber coelomocytes upon *V*. *splendidus* challenge. AjOTUB1 and circ-FGL1 were both identified to interact with AjMyc in the same region. In healthy sea cucumbers, circ-FGL1 is highly expressed, preferentially interacts with AjMyc, promotes AjMyc degradation, inhibits AjMyc nuclear translocation, and inhibits the AjBax-AjCyt c-mediated apoptosis pathway. In *V*. *splendidus*-challenged sea cucumbers, with a decrease in circ-FGL1 expression, induced AjOTUB1 preferentially interacts with AjMyc, prevents circ-FGL1-mediated AjMyc protein degradation, promotes AjMyc nuclear translocation, and enhances the AjBax-AjCyt c-mediated apoptosis pathway. The schematic was created with BioRender.com.

Our study provides valuable insights into the roles of circRNAs in *A*. *japonicus* challenged by *V*. *splendidus*- and presents evidence for the underlying mechanisms by which circRNAs participate in the regulation of *V*. *splendidus*-induced apoptosis. This work identified circ-FGL1 as a negative regulator of apoptosis and revealed that it displays outstanding ability in SUS progression during *V*. *splendidus* invasion. Mechanistically, circ-FGL1 and AjOTUB1 interact with the AjMyc protein in the same region, with circ-FGL1/AjMyc showing a higher affinity. In a healthy state, high levels of circ-FGL1 bind to AjMyc protein prior to promoting its degradation through ubiquitination. However, following *V*. *splendidus* infection, the induced expression of the AjOTUB1 protein leads to its preferential binding to the AjMyc protein, preventing its degradation. AjMyc is then transferred to the nucleus, where it promotes the transcription of AjCyt c/AjBax, ultimately inducing coelomocyte apoptosis (Fig 9). These findings offer potential new therapeutic targets for treating in echinoderms during bacterial invasion.

## Supporting information

S1 Fig The top 10 proteins pulled down by circ-FGL1 in coelomocytes and intestines. The red number represents the protein score identified by MS.
(TIF)

S2 Fig The protein expression levels of AjMyc-f1, AjMyc-f2, and AjMyc-f3 were investigated in HEK 293T cells transfected with circ-FGL1-f1, circ-FGL1-f2, or empty vectors, and AjMyc-f1, AjMyc-f2, or AjMyc-f3 using anti-FLAG antibodies as shown in Fig 4E. (TIF)

S3 Fig The protein expression levels of AjMyc-f1 were investigated in HEK 293T cells transfected with circ-FGL1-f1, circ-FGL1-f2, or empty vectors and AjMyc-f1, or AjMyc-f1-mut using anti-FLAG antibodies, as shown in Fig 4H. (TIF)

S4 Fig The mRNA levels of AjMyc in the cytoplasm or nucleus of coelomocytes at 0, 6, 12, and 24 h post *V*. *splendidus* infection were detected by qRT-PCR. Ajβ-actin and histone H3 served as the internal reference genes. The data are presented as the means ± SDs; n = 3. *P < 0.05 and **P < 0.01 indicate the significant differences.
(TIF)

S5 Fig AjMyc mRNA expression levels were detected in sea cucumber coelomocytes after treatment with the siRNAs of AjOTUB1 (siAjOTUB1), *A*. *japonicus* ubiquitin C-terminal hydrolase 7 (siAjUCH7), or siAjUCH14, respectively. Ajβ-actin served as the internal reference gene. The data are presented as the means ± SDs; n = 3. *P < 0.05 indicates a significant difference.
(TIF)

S6 Fig The protein expression levels of AjMyc in siAjOTUB1-, siAjUCH7-, and siAjUCH14-transfected coelomocytes were detected by western blotting. Ajβ-actin served as the control.
(TIF)

S7 Fig Cloning and characterization of AjOTUB1. Domain, nucleotide, and cDNA-derived aa sequences of AjOTUB1. The nucleotides shown in lowercase letters represent the UTRs, and the capital letters represent the ORFs. The predicted conserved Peptidase_C65 domain (35–266 aa) of AjOTUB1 is underlined in indigo.
(TIF)

S8 Fig Multiple alignments of the deduced amino acid sequences of AjOTUB1 with those of other known species of OTUB1s. A total of 50%, 75%, and 100% of the same aa residues were indigo, pink, and yellow, respectively, and white indicates less than 33% of the same amino acid residues. The amino acid sequences in the red box represent the Peptidase_C65 domain (35–266 aa).
(TIF)

S9 Fig The evolutionary relationship of AjOTUB1 with other known OTUB1 family members was analyzed using the N-J method. AjOTUB1 had a close relationship with OTUB1 from *Lytechinus variegatus*. (TIF)

S10 Fig The tertiary structures of OTUB1 in *A*. *japonicus*, *Patiria miniata*, *Asterias rubens*, *Homo sapiens*, *Myripristis murdjan*, and *Gallus gallus* were predicted by the SWISS-MODELModel/ExPASy program. (TIF)

S11 Fig The mRNA and protein levels of AjBax, Ajcaspase 3, and AjCyt c in *A*. *japonicus* coelomocytes or cultured primary coelomocytes treated with siAjOTUB1 and subjected to *V*. *splendidus* challenge or LPS stimulation were detected by qRT-PCR and western blotting. Ajβ-actin served as the control. The data are presented as the means ± SDs; n = 3. *P < 0.05, **P < 0.01, and ***P < 0.001 indicate the significant differences.
(TIF)

S1 Table Primers used in this study. (DOCX)

S2 Table Treatment groups of the interactions among FLAG-tagged AjMyc, circ-FGL1-f1 and EGFP-tagged AjOTUB1. (DOCX)

S1 Data Detailed information on the common circ-FGL1-interacting proteins in coelomocytes and intestines was identified by the RNA pull-down assay and MS analysis. (XLSX)

S2 Data The binding sites between circ-FGL1 and AjMyc and between AjOTUB1 and AjMyc (Blue), and the mutated nucleotides were marked in blue and underlined. (DOCX)
