# Supplementary figures and images for: Vibrio splendidus infection promotes circRNA-FGL1-regulated coelomocyte apoptosis via competitive binding to Myc with the deubiquitinase OTUB1 in Apostichopus japonicus
Source: PLoS Pathog. 2024 Aug 15;20(8):e1012463. doi: 10.1371/journal.ppat.1012463 (PMC11349225; doi:10.1371/journal.ppat.1012463)

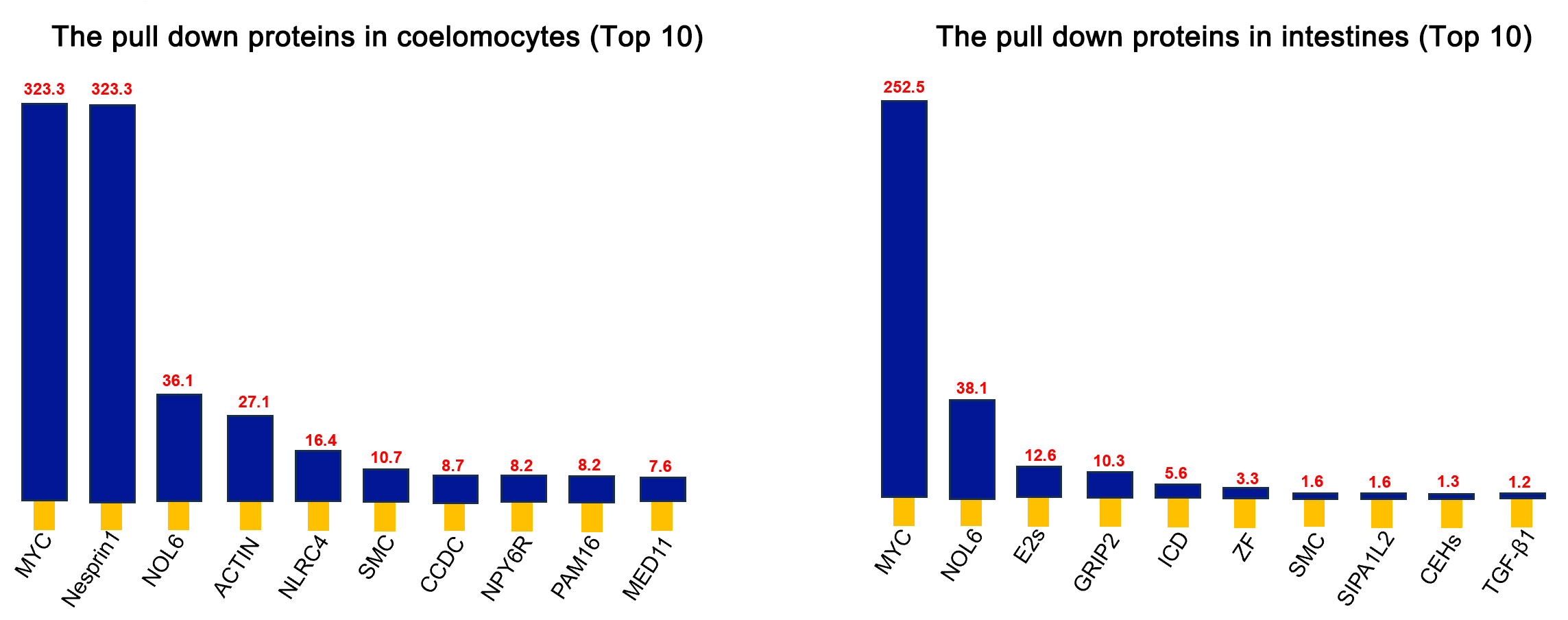

Supplement: S1 Fig — The red number represents the protein score identified by MS. (TIF) [file ppat.1012463.s001.tif]

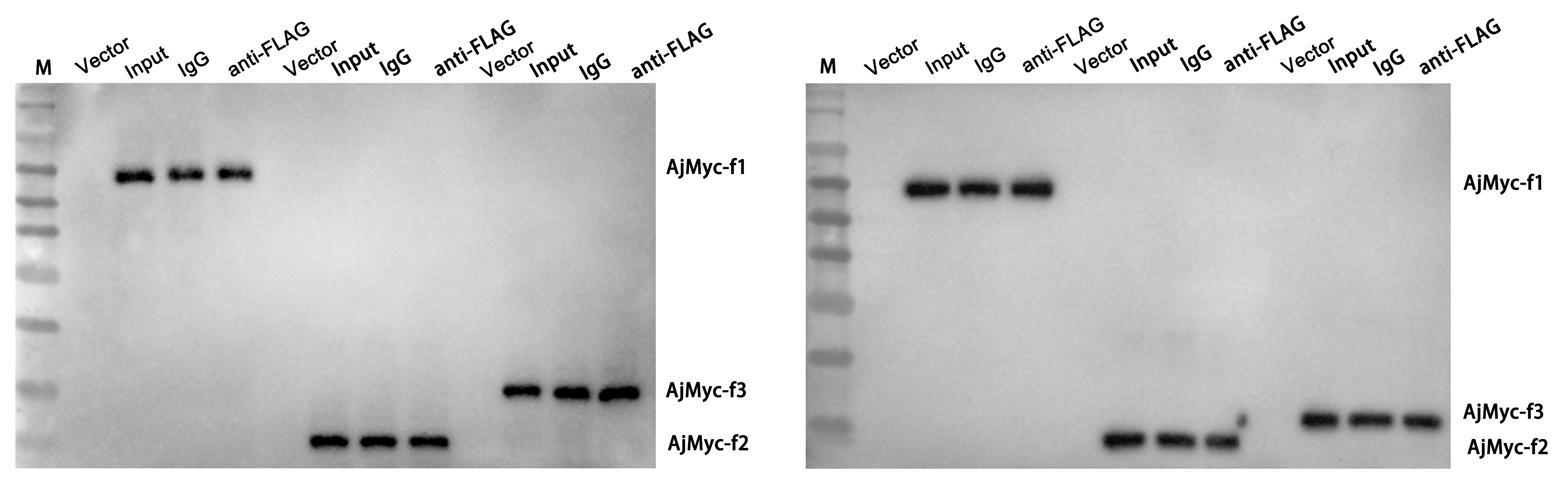

Supplement: S2 Fig — (TIF) [file ppat.1012463.s002.tif]

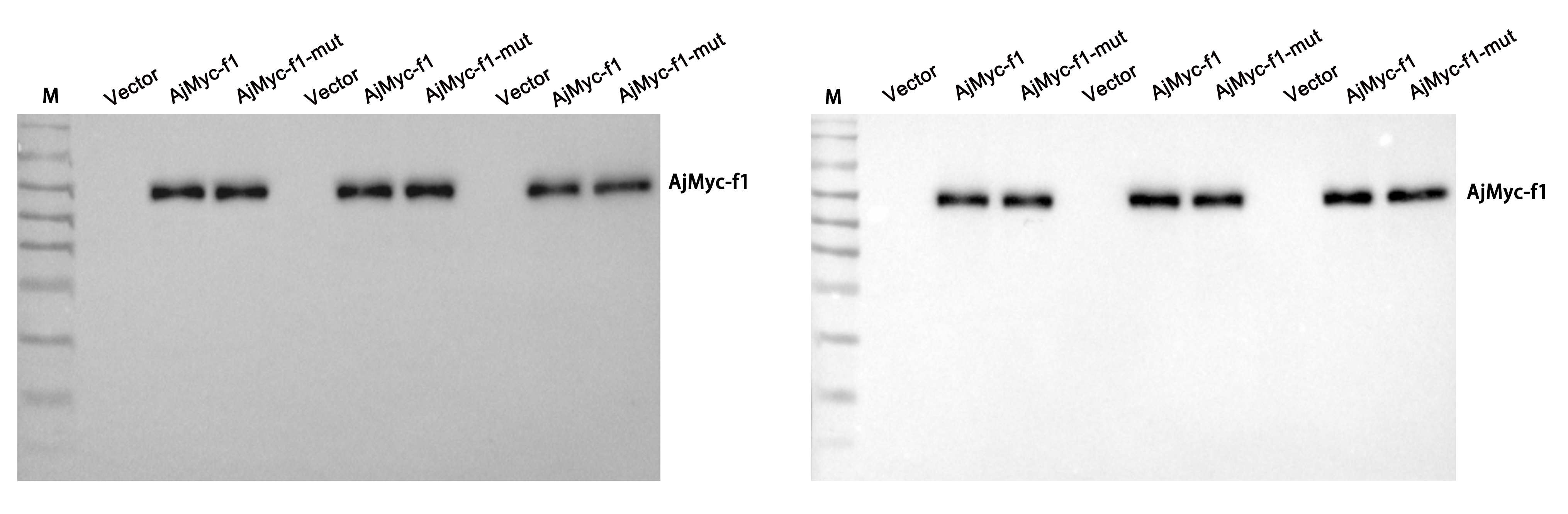

Supplement: S3 Fig — (TIF) [file ppat.1012463.s003.tif]

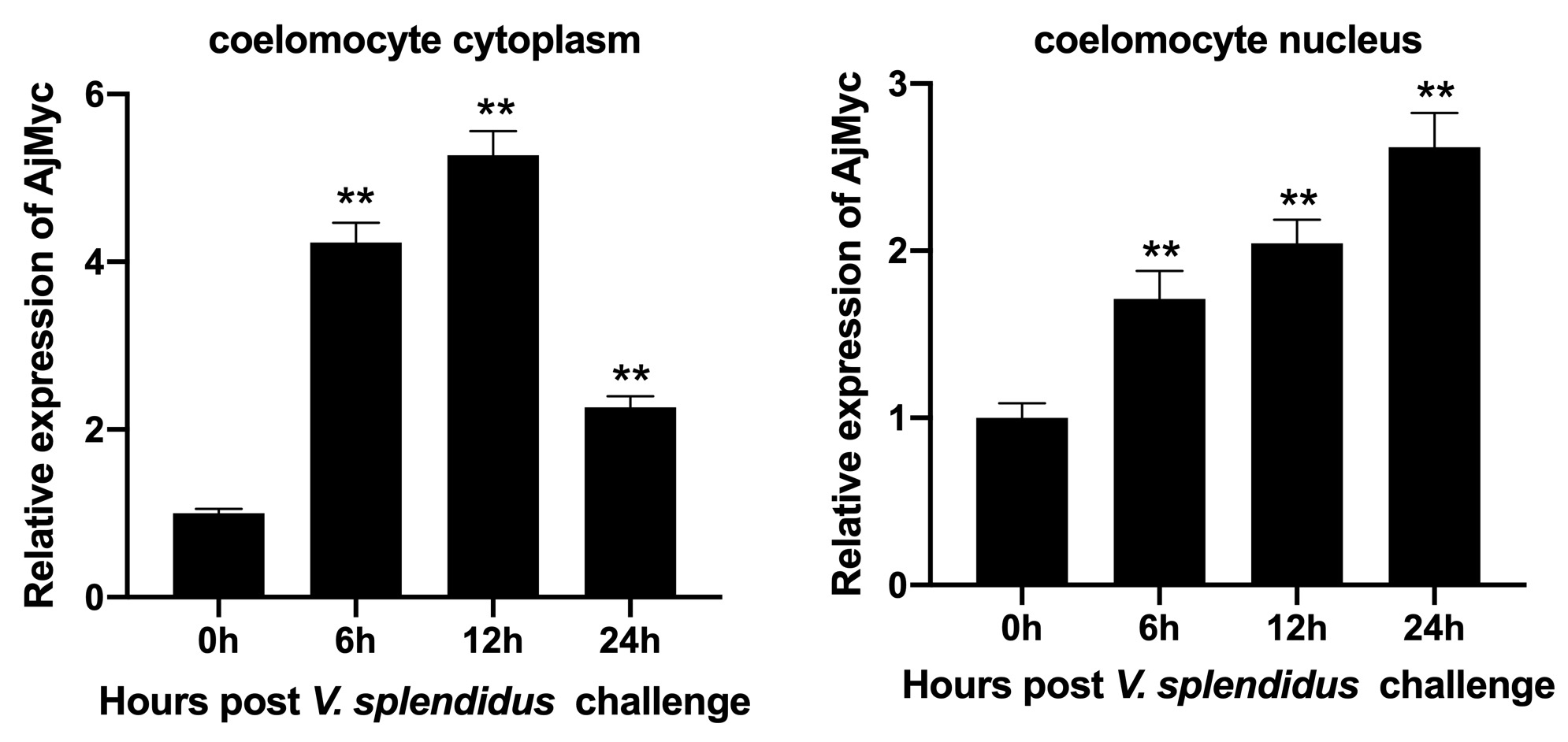

Supplement: S4 Fig — Ajβ-actin and histone H3 served as the internal reference genes. The data are presented as the means ± SDs; n = 3. *P < 0.05 and **P < 0.01 indicate the significant differences. (TIF) [file ppat.1012463.s004.tif]

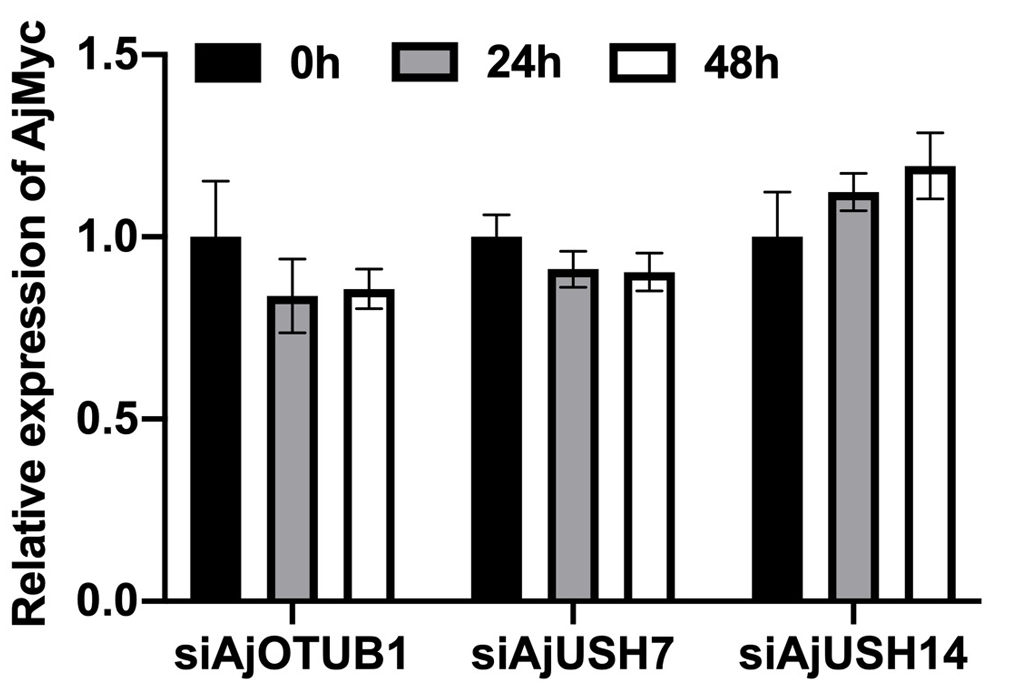

Supplement: S5 Fig — Ajβ-actin served as the internal reference gene. The data are presented as the means ± SDs; n = 3. *P < 0.05 indicates a significant difference. (TIF) [file ppat.1012463.s005.tif]

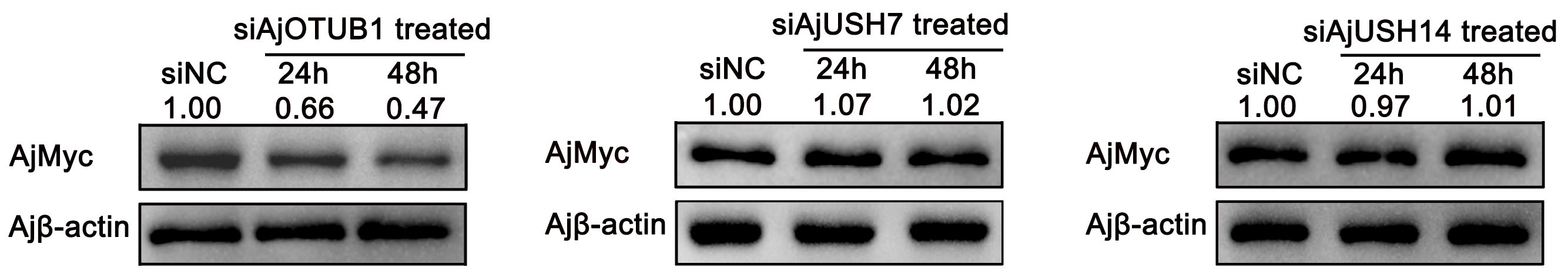

Supplement: S6 Fig — Ajβ-actin served as the control. (TIF) [file ppat.1012463.s006.tif]

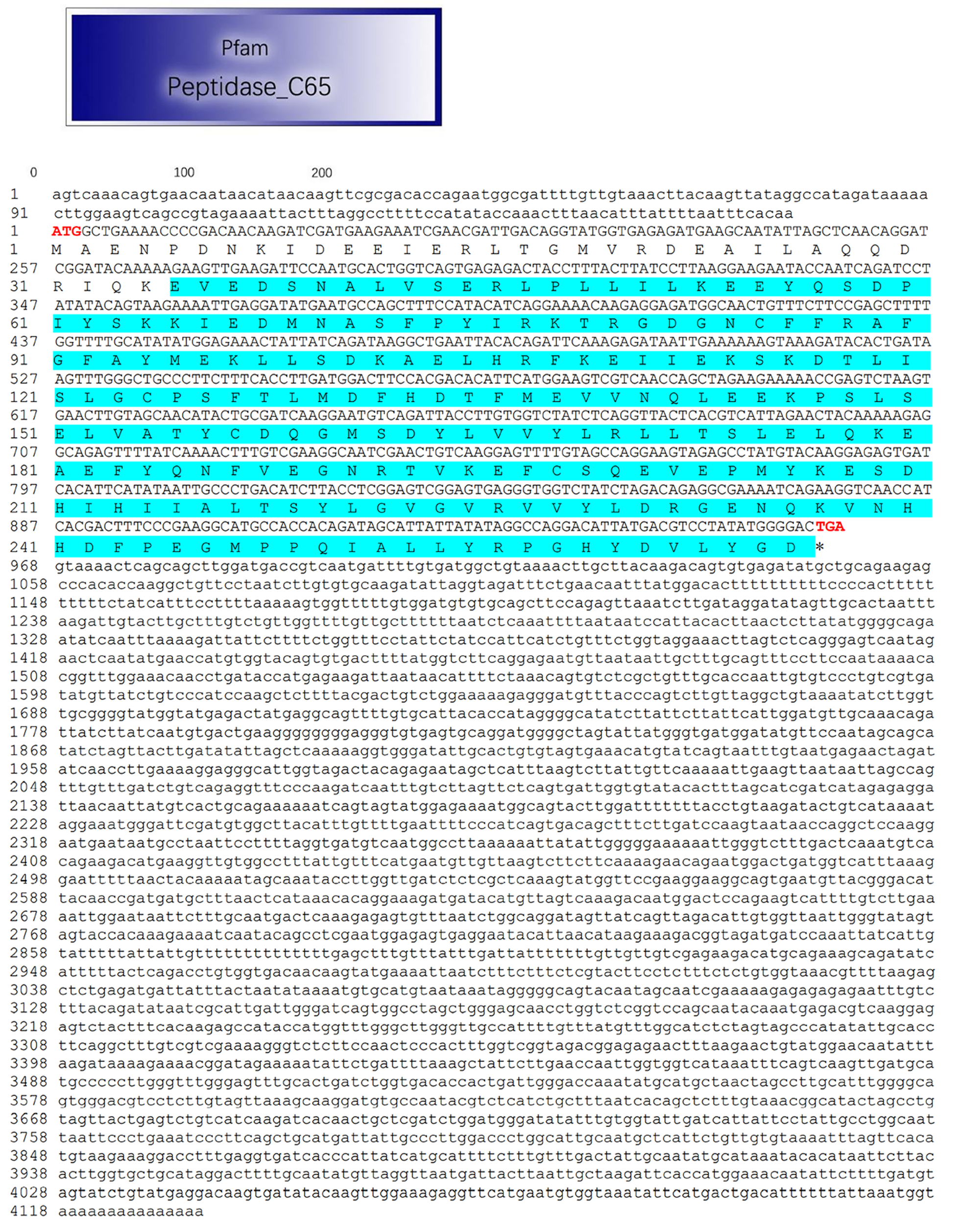

Supplement: S7 Fig — Domain, nucleotide, and cDNA-derived aa sequences of AjOTUB1. The nucleotides shown in lowercase letters represent the UTRs, and the capital letters represent the ORFs. The predicted conserved Peptidase_C65 domain (35–266 aa) of AjOTUB1 is underlined in indigo. (TIF) [file ppat.1012463.s007.tif]

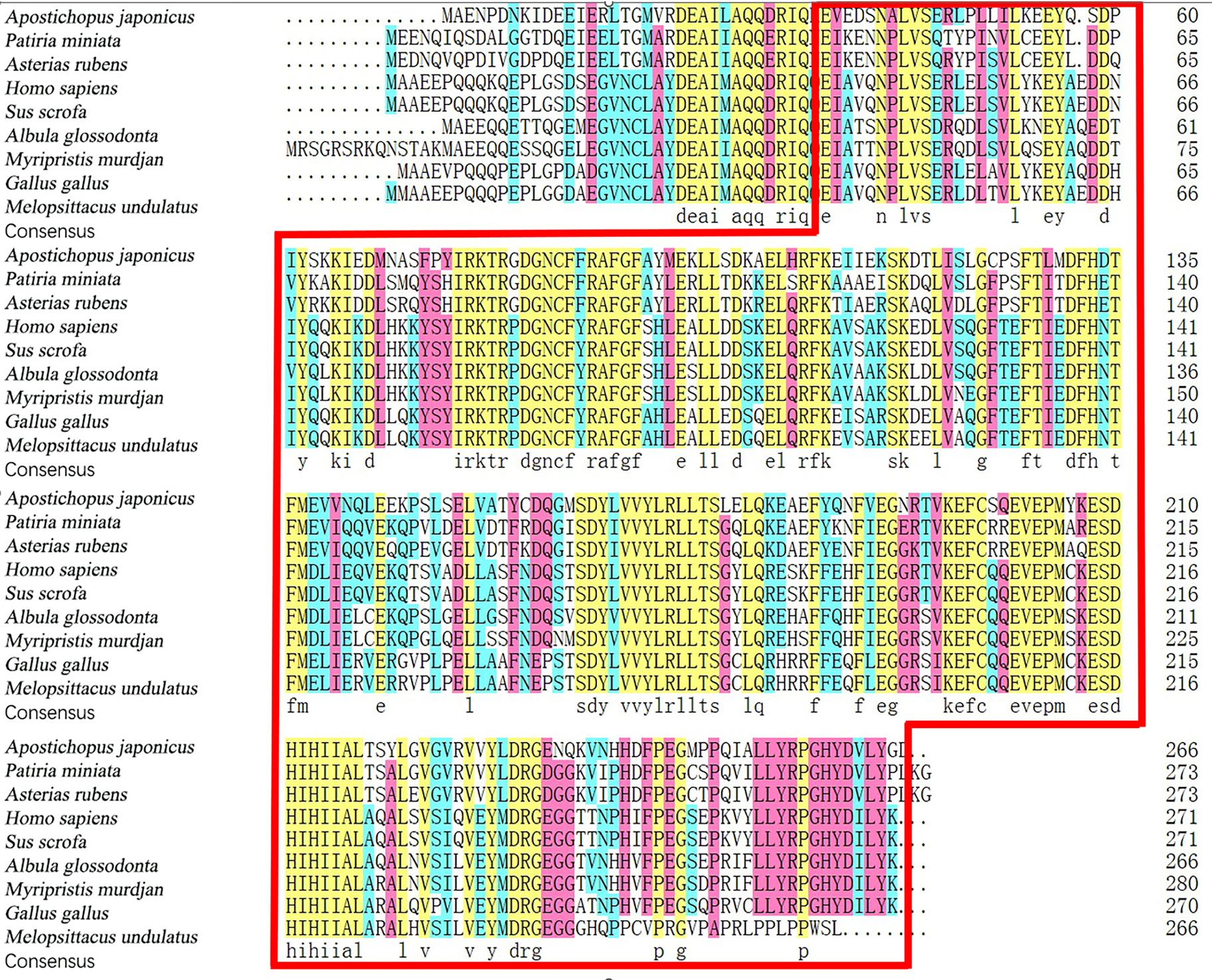

Supplement: S8 Fig — A total of 50%, 75%, and 100% of the same aa residues were indigo, pink, and yellow, respectively, and white indicates less than 33% of the same amino acid residues. The amino acid sequences in the red box represent the Peptidase_C65 domain (35–266 aa). (TIF) [file ppat.1012463.s008.tif]

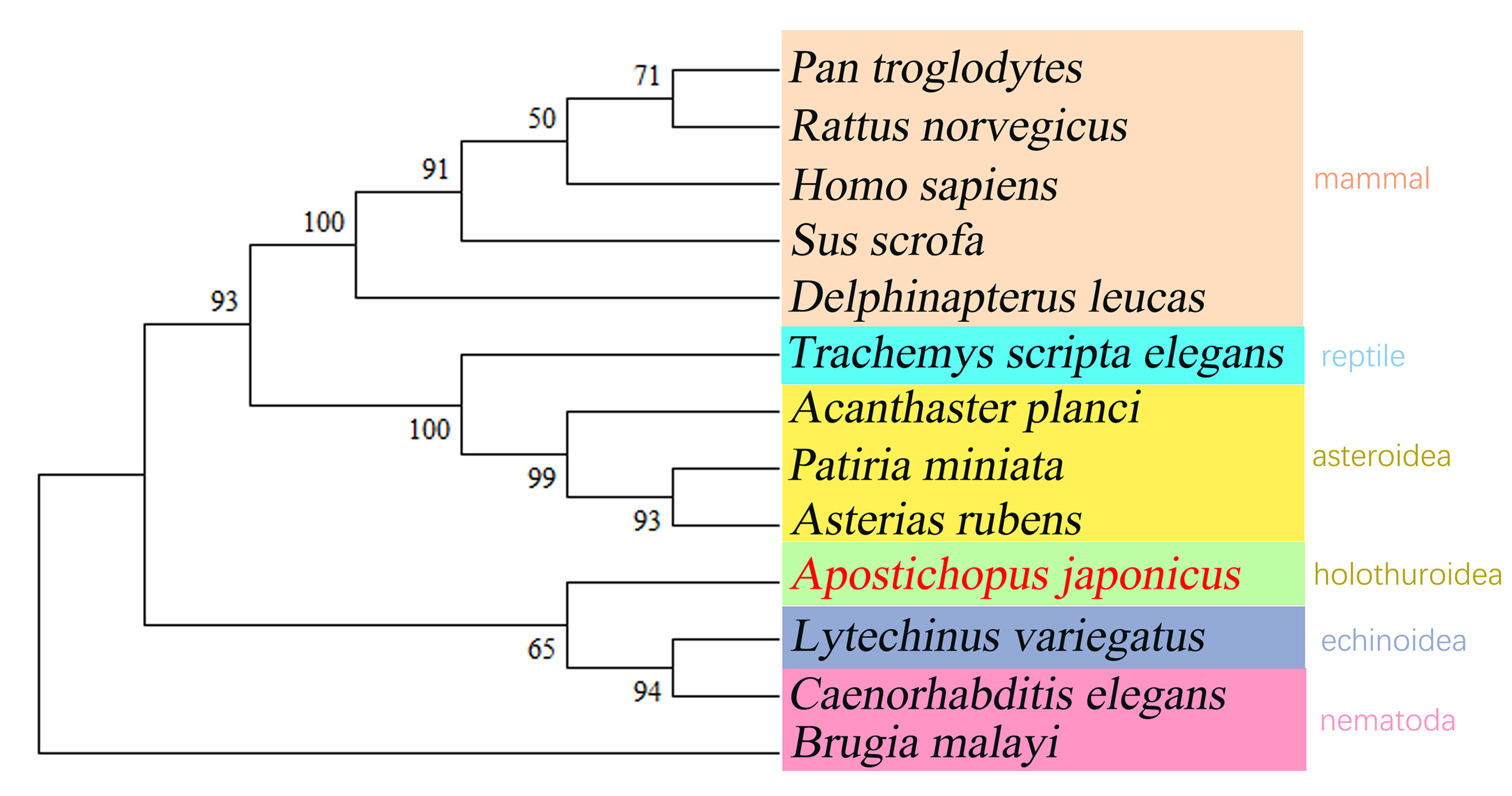

Supplement: S9 Fig — (TIF) [file ppat.1012463.s009.tif]

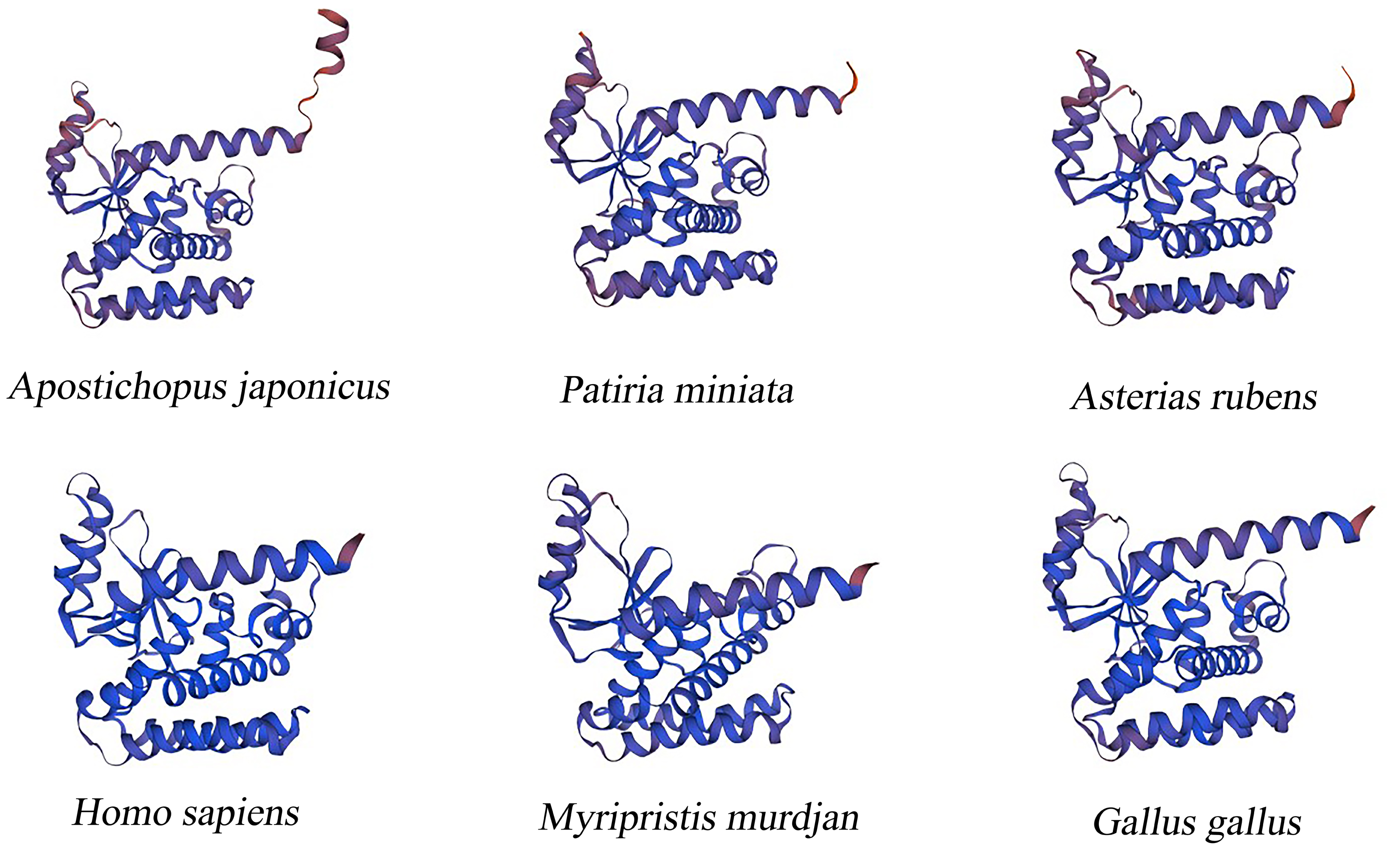

Supplement: S10 Fig — (TIF) [file ppat.1012463.s010.tif]

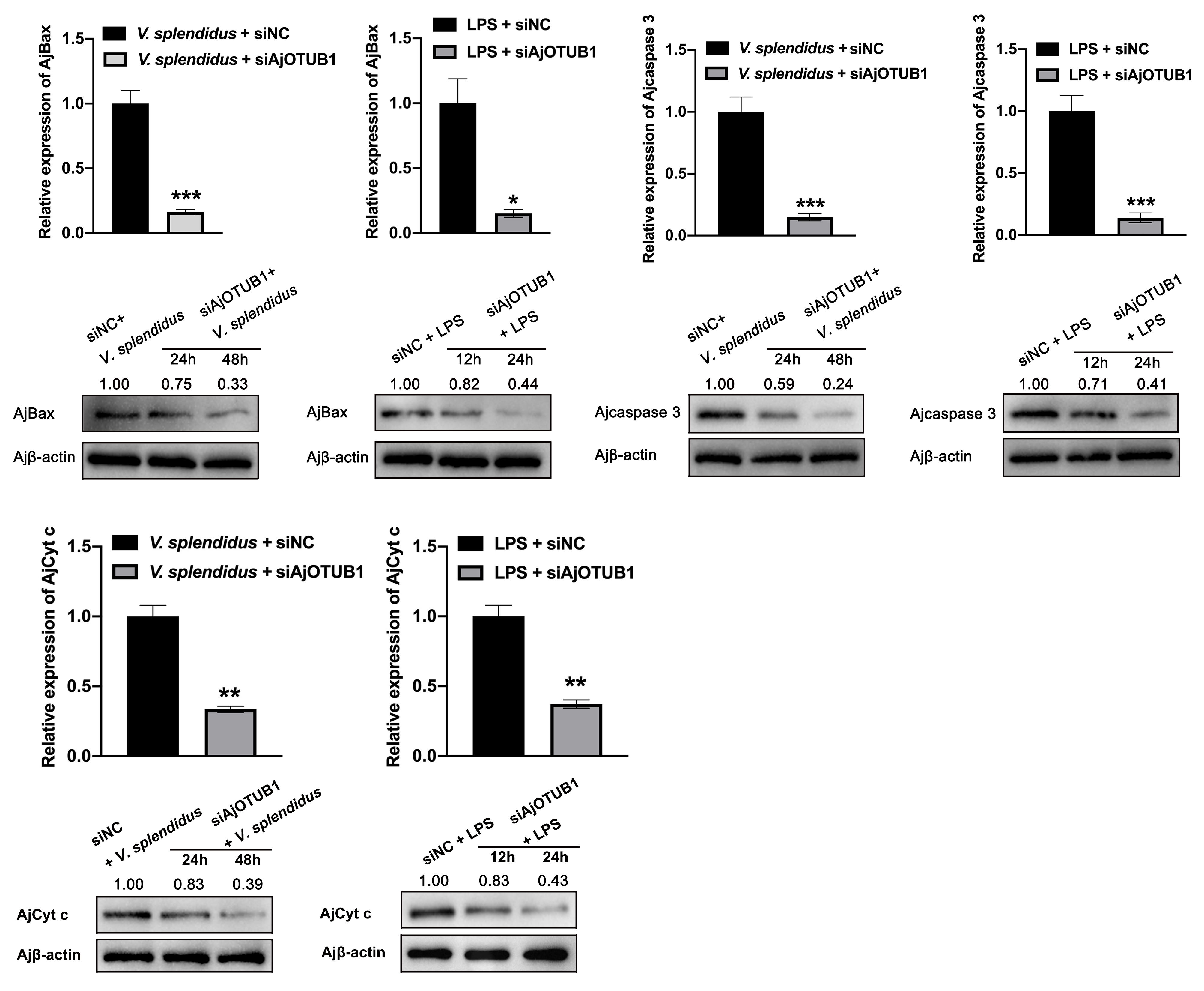

Supplement: S11 Fig — Ajβ-actin served as the control. The data are presented as the means ± SDs; n = 3. *P < 0.05, **P < 0.01, and ***P < 0.001 indicate the significant differences. (TIF) [file ppat.1012463.s011.tif]
