# Supplementary material for: Vibrio splendidus infection promotes circRNA-FGL1-regulated coelomocyte apoptosis via competitive binding to Myc with the deubiquitinase OTUB1 in Apostichopus japonicus
Source: PLoS Pathog. 2024 Aug 15;20(8):e1012463. doi: 10.1371/journal.ppat.1012463 (PMC11349225; doi:10.1371/journal.ppat.1012463)
Supplement: S1 Table — (DOCX) [file ppat.1012463.s012.docx]

**Table S1 Primers used in this study.**

| Primer Name | Primer Sequence (5'-3') | Used for |
| --- | --- | --- |
| circ-FGL1-div-F  circ-FGL1-div-R | CTTCAATTTCGTTTGACATT  AGGGAACGGCTCAAGATA | RT-PCR/ Sanger sequencing |
| circ-FGL1-con-F  circ-FGL1-con-R | GGCCTTCTTGTTTTCCTA  ACGCCTGTCATGATAATTACA | RT-PCR |
| q-circ-FGL1-F  q- circ-FGL1-R | CTTCAATTTCGTTTGACATT  AGGGAACGGCTCAAGATA | Real-time PCR |
| Ajβ-actin-F  Ajβ-actin-R | CCATTCAACCCTAAAGCCAACA  ACACACCGTCTCCTGAGTCCAT | Real-time PCR |
| AjFGL1-F  AjFGL1-R | ATGTTCTCTTTCATTATGAAAG  TGTTGTGGTCAGCGAGAGAATC | RT-PCR |
| qAjFGL1-F  qAjFGL1-R | CAGTCAGGTCTTTCGT  GTCCATCCTCCCTCGTCAGTT | Real-time PCR |
| si-circ-1-F  si-circ-1-R | AAGAGAGACGUCUGAAAGUTT  ACUUUCAGACGUCUCUCUUTT | RNA interference |
| si-circ-2-F  si-circ-2-R | GAGACGUCUGAAAGUUCUUTT  AAGAACUUUCAGACGUCUCTT | RNA interference |
| Ajcaspase 3-F  Ajcaspase 3-R | TTATTACTCTAAAAGAGAAGGACAA  GTACTTCTCTTGCAGTCTGTG | Real-time PCR |
| Ajcaspase 6-F  Ajcaspase 6-R | AGAATGAACAGGAGAGTCGGAAC  TGAGTGAGAAAAGCACACAGGAA | Real-time PCR |
| Ajcaspase 8-F  Ajcaspase 8-R | GGAGATGGACAGGCGTTCTTTAC  CGATACCGTCCTTGTGGAACTCT | Real-time PCR |
| circ432-F  circ432-R | CAGACTTCCCTGTGGTGA  TGGTGTATGGACTGGCTC | RT-PCR |
| circ-FGL1 probe | AAAAAGTAAGAAGAACTTTCAGACG | RNA pull-down |
| AjMyc-F  AjMyc-R | CACCCCTGGTGATGATATTTG  TTTTGGGTTTCACAGGAGCT | Real-time PCR |
| AjMyc-f1-F  AjMyc-f1-R | CTCCGGACTCTAGAAAGCTTATGACTTCAGTGTGTGCCTTG  TGGTCTTTGTAGTCCTCGAGCTTGTCATCGTCATCCTTGT | Vector construction |
| AjMyc-f2-F  AjMyc-f2-R | TCCGGACTCTAGAAAGCTTATGTCTTCACACTCTGATTCTGA  TGGTCTTTGTAGTCCTCGAGGGGACCTGGTTTAGCGTAAT | Vector construction |
| AjMyc-f3-F  AjMyc-f3-R | TCCGGACTCTAGAAAGCTTATGAATGCGTTGGAATCTTGGGA  TGGTCTTTGTAGTCCTCGAGTAACTTACTGTGATCCTTTC | Vector construction |
| circ-FGL1-f1-F  circ-FGL1-f1-R | GACTTTTTTTTTATACTTCAGAATTTGCACATTTCCACCGAT  ATTCTTTTCCTTGCTTCTTACACAATACGAAAAAATGTCACC | Vector construction |
| circ-FGL1-f2-F  circ-FGL1-f2-R | GACTTTTTTTTTATACTTCAGTCACCATTAAATTGAAGTTGC  ATTCTTTTCCTTGCTTCTTACCCCAGTGACTGGTTAACATGTTG | Vector construction |
| AjMyc-wt-F  AjMyc-wt-R | CTCCGGACTCTAGAAAGCTTATGACTTCAGTGTGTGCCTTG  TGGTCTTTGTAGTCCTCGAGCTTGTCATCGTCATCCTTGT | Vector construction |
| circ-FGL1-f1-wt-F  circ-FGL1-f1-wt-R | GACTTTTTTTTTATACTTCAGAATTTGCACATTTCCACCGAT  ATTCTTTTCCTTGCTTCTTACACAATACGAAAAAATGTCACC | Vector construction |
| circ-FGL1-f2-wt-F  circ-FGL1-f2-wt-R | GACTTTTTTTTTATACTTCAGTCACCATTAAATTGAAGTTGC  ATTCTTTTCCTTGCTTCTTACCCCAGTGACTGGTTAACATGTTG | Vector construction |
| si-UCH7-F  si-UCH7-R | GCCUCUUCUUUACCAAUAATT  UUAUUGGUAAAGAAGAGGCTT | RNA interference |
| si-UCH14-F  si-UCH14-R | GGAGUGUGGAGGAGAAAUATT  UAUUUCUCCUCCACACUCCTT | RNA interference |
| AjOTUB1-3′-1  AjOTUB1-3′-2 | GGCAGAGTTTTATCAAAACTTTGTC  GAAGGTCAACCATCACGACTTTCCCG | 3′-RACE |
| AjOTUB1-5′-1  AjOTUB1-5′-2 | CTTATCCTTAAGGAAGAATACCAATCA  TCGAACGATTGACAGGTATGGTGAGA | 5′-RACE |
| qAjOTUB1-F  qAjOTUB1-R | AAATTGAGGATATGAATGC  TGTCGTGGAAGTCCATCAAGG | Real-time PCR |
| si-AjOTUB1-F  si-AjOTUB1-R | CCACCACAGAUAGCAUUAUTT  AUAAUGCUAUCUGUGGUGGTT | RNA interference |
| AjOTUB1-f1-F  AjOTUB1-f1-R | ACTCTCGGCATGGACGAGCTGTACAAGATGGCTGAAAACCCCGACAA  GGGTTTAAACGGGCCCTCTAGATCAGTCCCCATATAGGACGTCA | Vector construction |
| AjOTUB1-f2-F  AjOTUB1-f2-R | ACTCTCGGCATGGACGAGCTGTACAAGATAATTGAAAAAAGTAAAGA  GGGTTTAAACGGGCCCTCTAGATCAATCACTCTCCTTGTACATAG | Vector construction |
| circ-FGL1-f1-probe | AATTACAATACGAAAAAATGTCACC | RNA pull-down |
| circ-FGL1-f2-probe | TGGTGACCCAGTGACTGGTTAACAT | RNA pull-down |
| qAjBax-F  qAjBax-R | GGAACCAAAGTCATCACCG  GAACGAAATCTCTCCCAAG | Real-time PCR |
| si-AjBax-F  si-AjBax-R | GGUUGGAUUUCUGUGACAATT  UUGUCACAGAAAUCCAACCTT | RNA interference |
| siNC-F  siNC-R | UUCUCCGAACGUGUCACGUTT  ACGUGACACGUUCGGAGAATT | Negative control for siRNA |
| qAjCyt c-F  qAjCyt c-R | CTGGCAAGCACAAACAGGGTC TCTTTGGGGTTGAGGAGGTAG | Real-time PCR |
| si-AjCyt c-F  si-AjCyt c-R | GCACCUGGUUUCUCCUACATT  UGUAGGAGAAACCAGGUGCTT | RNA interference |
| RNU6B | CGTGAAGCGTTCCATATTTTAA | Real-time PCR |
