# Supplementary material for: Vibrio splendidus infection promotes circRNA-FGL1-regulated coelomocyte apoptosis via competitive binding to Myc with the deubiquitinase OTUB1 in Apostichopus japonicus
Source: PLoS Pathog. 2024 Aug 15;20(8):e1012463. doi: 10.1371/journal.ppat.1012463 (PMC11349225; doi:10.1371/journal.ppat.1012463)
Supplement: S2 Table — (DOCX) [file ppat.1012463.s013.docx]

**Table S2 Treatment groups of the interactions among FLAG-tagged AjMyc, circ-FGL1-f1 and EGFP-tagged AjOTUB1.**

| **Group** | **Treatment** |
| --- | --- |
| A | circ-FGL1-f1 (3 μg) + rAjMyc-f1 (1 μg) + rAjOTUB1-f1 (3 μg) |
| B | circ-FGL1-f1 (3 μg) + rAjMyc-f1 (1 μg) + rAjOTUB1-f1-mut (3 μg) |
| C | circ-FGL1-f1-mut (3 μg) + rAjMyc-f1 (1 μg) + rAjOTUB1-f1 (3 μg) |
