# Supplementary material for: Vibrio splendidus infection promotes circRNA-FGL1-regulated coelomocyte apoptosis via competitive binding to Myc with the deubiquitinase OTUB1 in Apostichopus japonicus
Source: PLoS Pathog. 2024 Aug 15;20(8):e1012463. doi: 10.1371/journal.ppat.1012463 (PMC11349225; doi:10.1371/journal.ppat.1012463)
Supplement: S2 Data — (DOCX) [file ppat.1012463.s015.docx]

Circ-FGL1 (circ-FGL1-f1, 1100-1500 bp), AjMyc binding site in blue

AATTTGCACATTTCCACCGATAAGAGATCAGTTATTGAATATTGAACAAATCTCATTGATACCAGTGGTGTTTCCTTTTCCACTTTCGATCTTCAGCCGGGGGAAAAGGGTTCATTAGGAGGAGGGGAAGGGGACATATTCTTTGCATTCAGCCGGGGAAGACATGGGCTACTACTGAGGATGTACCTTCCTCTCCCCCCATTATACAAACTATTTGCTTGGAAAACCATATACTTAGAGCTTGATTTGCTGCCCACGGGGAAACTTTGTATCGAATCCAAGGGGCGCAACCGGCGGCGGGGAATGATCATATAGTTCTCCATGCGTATTACATCCATATTTTATGTTGAAATGCACCTTTCACAGATCACCTATATTGTGGTGACATTTTTTCGTATTGT

Circ-FGL1 (circ-FGL1-f1-mut,1100-1500 bp), mutated AjMyc binding site in blue and underlined

AATTTGCACATTTCCACCGATAAGAGATCAGTTATTGAATATTGAACAAATCTCATCGAGCTGTCAGGTGTTTCCTTTTCCACTTTCGATCTTCAGCCGGGGGAAAAGGGTTCATTAGGAGGAGGGGAAGGGGACATATTCTTTGCATTCAGCCGGGGACCTGTCTCCGTACTACTGAGGATGTACACGGGTAGATCCCCATTATACAAACTATTTGCTTGGAAAACCATATACTTAGAGCTTGCTCACCTTGGACGGGGGAAACTTTGTATCGAATCCAAGGCGTATGTATCGCGGCGGGGAATGATCATATAGTTCTCCATGCGTATTACAAGATCACGGATCGTTGAAATGCACCTTTCACAGATCACCTATATTGTGGTGACATTTTTTCGTATTGT

Circ-FGL1 (circ-FGL1-f2,3100-3400 bp), AjMyc binding site in blue

TCACCATTAAATTGAAGTTGCACGTTATAACTCGCGTTATGAACGTTACTATGTAACTGTTTCTGTCGGTTTATATAATATGCACAATGATAAAACATAAAATAAAAATAAACTAAAGAAAGAAAGAAATATCCCATGAGTTTAACTTTGTTGCTTGATGATCTTGGGGGCCATCCATCAGCGGATGTCGCGTCCTGATGTGTGTGTGTGTTTGTGGGGGGAGGGGTAAGAGGTGTCCTTCTCTGATTTTGCGTTCAGGATGTATTCGACTGCTATGGCAACATGTTAACCAGTCACTGGG

Circ-FGL1 (circ-FGL1-f2-mut,3100-3400 bp), mutated AjMyc binding site in blue and underlined

TCACCATTAAATTGAAGTTGCACGTTATAACTCGCGTTATGAACGTTACTATGTAACTGTTTCTGTCGGTTTATATAATATGCACAATGATAAAACATAAAATAAAAATAAACTAAAGAAAGAAAGAAATATCCCATGAGTTTAACTTTGTTGCTTGATGATCTTGGGGGCCATCCATCAGCGGATGTCGCGTCCTGATGTGTGTGTGTGTTTGTGGGGGGAGGGGTAAGAGGTGTCCTTCTCTGATTTTGCGTTCAGGATGTATTCGACTGCTATTTGCTGTGCAACCGCAGTCACTGGG

AjMyc (AjMyc-f1, 1-1293 bp), AjOTUB1 binding site in blue

ATGACTTCAGTGTGTGCCTTGAGAGTTGACTGTGGTCGTGCAGTCCCACCTATGGAGACGATTGAAGTGGAGGAATATGGCATTCCTGGCTGGTTAAGTGATTCGGGGGCCACCCCTGGTGATGATATTTGGACAAAGTTTGGACTAGAAGAAACTGAGAGTATTTACCCTACTCCACCTCTGAGCCCAGACAGTAAATCTGACATTGGGGACAAGGATTGGCATGAAACTAGGTCAGATTCAGGACTTGATTTTGGTGAAGACCAAATGTCTTGGCAACCAGAACATAGTGTAGACATTACACCTCTTCTCGAATTGGACTGTGAACTAGACATGTTAAAACCTATTCAATGTGCAGCTCCTGTGAAACCCAAAAGTGAACTGAAAGTGAGTTTGATACAAGATTGTATGTGGAGTGCTTACAAAAAGTTGCTACTGAAAGACAGTGAAAAACTGGATGTGAATCGTTCCAAAACAACGCAGGAGAACTTGTACCAAGAATTTAACCAACCCTCAGATTGTGTTGATCCAACAACAGTCTTTCCATACCCGTTATCAATGACAGAAACACATTTGGACCTCAACATAGGAAGCCAGTCGTCTTCACACTCTGATTCTGAAGAAGAAATTGATGTTGTCACCATAGCAACCATCGAGAGCACTCCTGAGGAAAAGAAAAGGTCTTCATCTTCCACCACCAAGAAGGACTCTCACAAGCACAAACACAGGCGCATGTACGAGGTGTCTAATCCAGAGCATGATTACGCTAAACCAGGTCCCAAACATTTGAGCAAACTTGGTAGGTCACTAAAGCGGAGCAAGAGTTCTTGTTCGTCACTATCGCACAGTCTCGTGAAGAGAAAAAGGATGAACGAAGGAGTAAATCCCATAGCACTAAAGAATGCGTTGGAATCTTGGGATCCAAAATCGAGGGGTCTTGGTAGCATGTACAACCCAGCAAGTAGCCGTGGTAGTAGTAAAAACAGTAGCAGGTGTTCTAGTCAAAGTGGTAGCCACTCTGCATCAGACTCTGAAGACTGTGACAAGAGAGTCAATCACAACGTTTTGGAAAGGAAGCGAAGAGAAGACCTCCGCAGCGCGTTCTTTAGGTTACGAGATCGCATCCCAGAGCTTGAATCAAAGGAACGTGCCTCCAAAGTAGTCATTTTAGAAAAATCAAGGACCTATGTTATGTCACTGAGAAAGGATCACAGTAAGTTAGCTCAGGAGAAGGAAATTCAGGCCCGACGGCACAAGGAACTGTTAGCCCGGCTCAGGCAGCTGAGGCAACGGTGA

AjMyc (AjMyc-f1-mut, 1-1293 bp), mutated AjOTUB1 binding site in blue and underlined

ATGACTTCAGTGTGTGCCTTGAGAGTTGACTGTGGTCGTGCAGTCCCACCTATGGAGACGATTGAAGTGGAGGAATATGGCATTCCTGGCTGGTTAAGTGATTCGGGGGCCACCCAGCGTCTGCATGCATGGACAAAGTTTGGACTAGAAGAAACTGAGAGTATTTACCCTACTCCACCTCTGAGCCCAGACAGTAAATCTGACATTGGGGACAAGGATTGGCATGAAACTAGGTCAGATTCAGGACTTGATTTTGGTGAAGACCAAATGTCTTGGCAACCAGAACATAGTGTAGACATTACACCTCTTCTCGAATTGGACTGTGAACTAGACATGTTAAAACCTATTCAATGTGCAGCTCCTGTGAAACCCAAAAGTGAACTGAAAGTGAGTTTGATACAAGATTGTATGTGGAGTGCTTACAAAAAGTTGCTACTGAAAGACAGTGAAAAACTGGATGTGAATCGTTCGTCGTCAACCTTCAAGAACTAGTCGGTAGAATTTAACCAACCCTCAGATTGTGAGTCACCTCCAACAGTCTTTCCATACCCGTTATCAATGACAGAAACACATTTGGACCTCAACATAGGAAGCCAGTCGTCTTCACACTCTGATTCTGAAGAAGAAATTGATGTTGTCACCATAGCAACCATCGAGAGCACTCCTGAGGAAAAGAAAAGGTCTTCATCTTCCACCACCAAGAAGGACTCTCACAAGCACAAACACAGTCGCCACGTGGTCAGCTCTAATCCAGAGCATGATTACTCTTTCGTTGGTCAGTCGTTAGCGAGCAAACTCTCGTCCAGACTAAAGCGGAGCATCTGTAGACCGTCACCACTGATCTACAGTCTCGTGAAGAGAAAAAGGATGAACGAAGGAGTAAATCCCATAGCACTAAAGAATGCGTTGGAATCTTGGGATCCAAAATCGAGGGGTCTTGGTAGCATGTACAACCCAGCAAGTAGCCGTGGTAGTAGTAAAAACAGTAGCAGGTGTTCTAGTCAAAGTGGTAGCCACTCTGCATCAGACTCTGAAGACTGTGACAAGAGAGTCAATCACAACGTTTTGGAAAGGAAGCGAAGAGAAGACCTCCGCAGCGCGTTCTTTAGGTTACGAGATCGCATCCCAGAGCTTGAATCAAAGGAACGTGCCTCCAAAGTAGTCATTTTAGAAAAATCAAGGACCTATGTTATGTCACTGAGAAAGGATCACAGTAAGTTAGCTCAGGAGAAGGAAATTCAGGCCCGACGGCACAAGGAACTGTTAGCCCGGCTCAGGCAGCTGAGGCAACGGTGA

AjMyc (AjMyc-f3, 900-1221 bp), AjOTUB1 binding site in blue

GAATGCGTTGGAATCTTGGGATCCAAAATCGAGGGGTCTTGGTAGCATGTACAACCCAGCAAGTAGCCGTGGTAGTAGTAAAAACAGTAGCAGGTGTTCTAGTCAAAGTGGTAGCCACTCTGCATCAGACTCTGAAGACTGTGACAAGAGAGTCAATCACAACGTTTTGGAAAGGAAGCGAAGAGAAGACCTCCGCAGCGCGTTCTTTAGGTTACGAGATCGCATCCCAGAGCTTGAATCAAAGGAACGTGCCTCCAAAGTAGTCATTTTAGAAAAATCAAGGACCTATGTTATGTCACTGAGAAAGGATCACAGTAAGTTA

AjMyc (AjMyc-f3-mut, 900-1221 bp), AjOTUB1 binding site in blue and underlined

GAATGCGTTGGAATCTTGGGATCCAAAATCGAGGGGTCTTGGTAGCATGTACAACCCAGCAAGTAGCCCACCATCCTCGTTCCTGTCACGCAGGTGTTCTAATGTCTTCCCATCCCACTCTGCATCAGTGAGGCTTATTACTGACAAGAGAGTCAATCACAACGTTTTGGAAAGGAAGCTCCCGCTGCTGGAAGGTTATCAAGTCTTTAGGTGCAATCGGACTTGCCCAGAGCTTGAATCAAAGGAACGTGCCTCCAAAGTAGTCATTTTAGAAAAATCAAGGACCTATGTTATGTCACTGAGAAAGGATCACAGTAAGTTA

AjOTUB1 (AjOTUB1-f1, 1-801 bp), AjMyc binding site in blue

ATGGCTGAAAACCCCGACAACAAGATCGATGAAGAAATCGAACGATTGACAGGTATGGTGAGAGATGAAGCAATATTAGCTCAACAGGATCGGATACAAAAAGAAGTTGAAGATTCCAATGCACTGGTCAGTGAGAGACTACCTTTACTTATCCTTAAGGAAGAATACCAATCAGATCCTATATACAGTAAGAAAATTGAGGATATGAATGCCAGCTTTCCATACATCAGGAAAACAAGAGGAGATGGCAACTGTTTCTTCCGAGCTTTTGGTTTTGCATATATGGAGAAACTATTATCAGATAAGGCTGAATTACACAGATTCAAAGAGATAATTGAAAAAAGTAAAGATACACTGATAAGTTTGGGCTGCCCTTCTTTCACCTTGATGGACTTCCACGACACATTCATGGAAGTCGTCAACCAGCTAGAAGAAAAACCGAGTCTAAGTGAACTTGTAGCAACATACTGCGATCAAGGAATGTCAGATTACCTTGTGGTCTATCTCAGGTTACTCACGTCATTAGAACTACAAAAAGAGGCAGAGTTTTATCAAAACTTTGTCGAAGGCAATCGAACTGTCAAGGAGTTTTGTAGCCAGGAAGTAGAGCCTATGTACAAGGAGAGTGATCACATTCATATAATTGCCCTGACATCTTACCTCGGAGTCGGAGTGAGGGTGGTCTATCTAGACAGAGGCGAAAATCAGAAGGTCAACCATCACGACTTTCCCGAAGGCATGCCACCACAGATAGCATTATTATATAGGCCAGGACATTATGACGTCCTATATGGGGACTGA

AjOTUB1 (AjOTUB1-f1-mut,1-801 bp), mutated AjMyc binding site in blue and underlined

ATGGCTGAAAACCCCGACAACAAGATCGTATCGGCAATGCTATCTTTGACAGGTATGGTCTGACCAGTAGCAATATTAGCTCATGCGTCGTAGATACAAAAAGAAGTTGAAGATTCCAATGCACTGGTCAGTGAGAGACTACCTTTACTTATCCTTAAGGAAGAATACCAATCAGATCCTATATACAGTAAGAAAATTGAGGATATGAATGCCAGCTTTCCATACATCAGGAAAACAAGAGGAGATGGCAACTGTTTCTTCCGAGCTTTTGGTTTTGCATATATGGAGAAACTATTATCAGATAAGGCTGAATTACACAGATTCAAAGAGATAATTGAAAAAAGTCCACTCTCACTGATAAGTTTGGGCTGCCCTTCTTTCACCTTGATGGACTTCCACGACACATTGTTGTGTCTCGTCAACCAGCTAGAAGAAAAACCGAGTCTAAGTGAACTTGTAGCAACATACTGCGATCAAGGAATGAGACTACGTGTCGTGGTCTATCTCAGGTTACTCACGTCATTAGAACTACAAAAAGAGGCAGAGTTTTATCAAAACTTTGTCGAAGGCAATCGAACTGTCAAGGAGTTTTGTAGCTGGCCTACAGAGCCTATGTACGAGTCTCAAGATCACATTCATATAATTGCCCTGACATCTTACCTCGGAGTCGGAGTGAGGGTGGTCTATCTAGACAGAGGCGAAAATCAGAAGGTCAACCATCACGACTTTCCCGAAGGCATGCCACCACAGATAGCATTATTATATAGGCCAGGACATTATGACGTCCTATATGGGGACTGA
